# Bamboo Shark as a Small Animal Model for Single Domain Antibody Production

**DOI:** 10.3389/fbioe.2021.792111

**Published:** 2021-12-08

**Authors:** Likun Wei, Meiniang Wang, Haitao Xiang, Yuan Jiang, Jinhua Gong, Dan Su, M. A. R. Al Azad, Hongming Dong, Limin Feng, Jiajun Wu, Leo Lai Chan, Naibo Yang, Jiahai Shi

**Affiliations:** ^1^ State Key Laboratory of Marine Pollution, Department of Biomedical Sciences, City University of Hong Kong, Hong Kong, China; ^2^ BGI-Shenzhen, Shenzhen, China; ^3^ China National GeneBank, BGI-Shenzhen, Shenzhen, China; ^4^ College of Life Sciences, University of Chinese Academy of Sciences, Beijing, China; ^5^ Shen Zhen Research Institute, City University of Hong Kong, Shen Zhen, China; ^6^ Complete Genomics Inc., San Jose, CA, United States; ^7^ Tung Biomedical Sciences Centre, City University of Hong Kong, Hong Kong, China; ^8^ Synthetic Biology Translational Research Programme, Yong Loo Lin School of Medicine, National University of Singapore, Singapore, Singapore; ^9^ Department of Biochemistry, Yong Loo Lin School of Medicine, National University of Singapore, Singapore, Singapore

**Keywords:** single domain antibody, bamboo shark, IgNAR, immunization, vNAR, immune repertoire

## Abstract

The development of shark single domain antibodies (sdAbs) is hindered by the high cost and tediousness of large-sized shark farming. Here, we demonstrated white-spotted bamboo sharks (*Chiloscyllium plagiosum*) being cultivated commercially as a promising small animal model to produce sdAbs. We found that immunoglobulin new antigen receptor (IgNAR) presented in bamboo shark genome, transcriptome, and plasma. Four complete IgNAR clusters including variable domains (vNARs) were discovered in the germline, and the Variable–Joining pair from IgNAR1 cluster was dominant from immune repertoires in blood. Bamboo sharks developed effective immune responses upon green fluorescent protein (GFP), near-infrared fluorescent protein iRFP713, and Freund’s adjuvant immunization revealed by elevated lymphocyte counts and antigen specific IgNAR. Before and after immunization, the complementarity determining region 3 (CDR3) of IgNAR were the major determinant of IgNAR diversity revealed by 400-bp deep sequencing. To prove that bamboo sharks could produce high-affinity IgNAR, we isolated anti-GFP and anti-iRFP713 vNARs with up to 0.3 and 3.8 nM affinities, respectively, from immunized sharks. Moreover, we constructed biparatopic vNARs with the highest known affinities (20.7 pM) to GFP and validated the functions of anti-GFP vNARs as intrabodies in mammalian cells. Taken together, our study will accelerate the discovery and development of bamboo shark sdAbs for biomedical industry at low cost and easy operation.

## Introduction

Single domain antibodies (sdAbs) are preferred over traditional antibodies because of their simple architecture, high thermal and chemical stability, good solubility, high tissue penetration, easy modularity, and straightforward expression in *Escherichia coli* (Holliger and Hudson, 2005; Simmons et al., 2006; Koenning et al., 2017; Steven et al., 2017; Ubah et al., 2017; Ubah et al., 2020; Stocki et al., 2021). SdAbs are derived from the antigen-binding variable domain (VHH) of camel heavy chain–only antibody or the variable domain (vNAR) of cartilaginous fish immunoglobulin new antigen receptor (IgNAR) (Koenning et al., 2017). As the most ancient vertebrates, cartilaginous fish have three immunoglobulin (Ig) isotypes, namely, IgM, IgW, and IgNAR (Diaz et al., 2002; Dooley and Flajnik, 2005; Matz et al., 2021). IgM and IgNAR are the major antibodies involved in adaptive humoral immune response.

In the same manner as VHH production (Holliger and Hudson, 2005), vNARs from immunized libraries (Camacho-Villegas et al., 2013; Kovaleva et al., 2017; Leow et al., 2018) generally have higher affinities and specificities than those from semi-synthetic libraries (Häsler et al., 2016; Cabanillas-Bernal et al., 2019; Stocki et al., 2021) and naïve libraries (Simmons et al., 2006; Feng et al., 2019). This result is because IgNARs have experienced iterative affinity maturation in immunized sharks upon immunization (Dooley et al., 2006). Immunized libraries have been reported so far only in nurse shark (Dooley, 2003), ornate wobbegong (Leow et al., 2018), and horn shark (Bojalil et al., 2013). However, most shark species used for vNAR generation are difficult to maintain in captivity because of their endangered state, large body size, slow maturity, aggressive temper, or fast movement (Dooley, 2003; Bojalil et al., 2013; Leow et al., 2018). Moreover, some cartilaginous fish do not have adaptive humoral immune response, such as small-spotted catshark (Crouch et al., 2013a). The white-spotted bamboo sharks *Chiloscyllium plagiosum* are a small inshore demersal shark species (up to 1 m of adult length) (Chen and Liu, 2006) that are commonly found in home aquariums. They are sedentary, harmless, robust, and fast to mature (less than 5 years) (Smith et al., 2004). They can be bred artificially and domesticated to eat artificial feed (Chen and Liu, 2006), making them suitable for large-scale husbandry.

To date, only a semi-synthetic library (CDR3 randomization) was constructed from bamboo shark by Kolmar’s team, but the resulted vNAR binders usually require *in vitro* affinity maturation to improve their affinities (Zielonka et al., 2014; Macarrón Palacios et al., 2020). However, whether or not bamboo sharks have a functional adaptive humoral IgNAR immune response remains unclear. Furthermore, whether or not high-affinity functional vNARs can be isolated from immunized bamboo sharks is unknown. The construction of a high-diversity immunized library requires detailed information on genes encoding for antibodies. IgNARs present as multiple clusters in the shark genome (Diaz et al., 2002). The loci for IgNARs have been recently identified within the white-spotted bamboo shark genome representing the only chromosome-level cartilaginous fish genome (Zhang et al., 2020a). The whole genome of brownbanded bamboo shark (*Chiloscyllium punctatum*) has also been reported but lacks an immunology-related analysis (Hara et al., 2018). The detailed germline configuration of IgNAR clusters in whitespotted or brownbanded bamboo shark remains to be studied.

Here, our data showed that bamboo shark genome had seven IgNAR clusters, in which only four of them (IgNAR1, 2, 5, and 6) had variable domain genes. Multi-tissue transcriptomes revealed that IgNAR1, 2, and 6 were expressed in blood and spleen, and IgNAR, 1, and 2 were preferentially expressed in adult sharks. Meanwhile, we discovered that IgNAR_short_ transmembrane form, IgNAR_long_ secretory form, and multiple IgNAR secretory multimers (e.g., trimer, tetramer, and pentamer) presented in the blood of bamboo sharks. Being immunized with green fluorescent protein (GFP), near-infrared fluorescent protein iRFP713, and Freund’s adjuvant, bamboo sharks developed effect humoral IgNAR responses validated by increased lymphocyte counts and an enhanced antigen specific IgNAR level. High-throughput sequencing revealed that the Shannon index of CDR3 amino acid decreased along with the immunization progression, and on the contrary, the cumulative frequency of top 100 clones increased, indicating the expansion of high-frequency clonetypes. We also observed the presence of strong and rapid IgNAR recall response upon reencounter with antigens. Among four different immunization strategies, the biweekly subcutaneous antigen administration was a proper way for bamboo sharks to get high immune efficacy. Furthermore, anti-GFP and anti-iRFP713 vNARs with up to subnanomolar affinities were generated from immunized bamboo sharks and anti-GFP vNARs were validated for use as intrabodies in mammalian cells and as biparatopic constructs with picomolar-binding affinities. Overall, our study demonstrates that bamboo shark can serve as a promising small animal model for high-affinity sdAb generation.

## Materials and Methods

### Animal Maintenance, Immunization, and Blood Collection

Wild-caught adult bamboo sharks (*C. plagiosum*) from the coastal area of Xiamen City, China, were used in this study. During the immunization period of this study, these animals were maintained at 23°C in natural seawater in small aquariums at the Aquatic Science Laboratory, City University of Hong Kong. All animal-related experiments were performed in accordance with protocols approved by the Department of Health of Hong Kong (Ref. No.: (18-159) in DH/SHS/8/2/5 Pt.4) and BGI Bioethics Committee (Ref. No.: No. FT 19032). The sharks were anesthetized with MS-222 (0.1 g/L seawater) before immunization and blood collection.

Three individual bamboo sharks in each immunization program (see details in Figure 2B) were immunized with GFP and iRFP713 simultaneously at each injection time. Complete Freund’s Adjuvant (CFA) or Incomplete Freund’s Adjuvant (IFA) was used as an immunopotentiator and prepared as an antigen emulsion (antigen: Freund’s Adjuvant = v:v = 1:1). Then 200 μg antigens (85 μg GFP and 115 μg iRFP713) were injected with CFA in the first immunization and 100 μg antigens (42.5 μg GFP and 57.5 μg iRFP713) for subsequent boosters with IFA. Multiple injection spots, such as four subcutaneous injection positions, were used to avoid tissue damage. Antigens and adjuvants in the subcutaneous delivery programs were separately injected in two times (the second to last and the fourth to last) to strengthen antibody response to native antigens. For the intravenous delivery program, the adjuvant was used only in the primary injection and abandoned in the subsequent boosters because of the toxicity of the adjuvant.

Blood was collected 2 weeks after each injection. 2 ml whole blood was drawn from the caudal vein and centrifuged at 300 g for 5 min at room temperature to separate buffy coat containing peripheral blood mononuclear cells (PBMC) and plasma for subsequent analysis. The buffy coat pellets were resuspended in TRIzol (Invitrogen, #15596026) before storage at −80°C, and the plasma samples were directly stored at −80°C.

### Complete Blood Count

All tests were performed manually, including packed cell volume (PCV), red blood cell (RBC) count, white blood cell (WBC) count, WBC differential (e.g., neutrophil, eosinophil, heterophil, monocyte, and lymphocyte), thrombocyte count, total plasma protein concentration, and hemoglobulin concentration. PCV was obtained by hematocrit tubes. Blood cells were counted in a Neubauer hemocytometer. The blood films were stained with Wright–Giemsa (Siemens Hematek 3000 System), and the white blood cell differential was read at 100× oil objective. Cells were identified according to published images (Arnold, 2005). Total plasma protein concentration was determined with a refractometer (Lines and Raine, 1970). Hemoglobin quantitation was performed using the sodium lauryl sulfate method (Karsan et al., 1993). Three shark individuals in each immunization program were involved in the above analyses.

### IgNAR Level

An ELISA was used to quantify the antigen specific IgNAR level. In brief, 96-well plates were coated with antigens (100 ng GFP or iRFP713 in 100 μl PBS) in each well at 4°C overnight and then blocked with 5% BSA-PBS at room temperature (RT) for 3 h. Uncoated wells were set for controls and conducted with the same process. Plasma was diluted 1:100 in 3% BSA-PBS and introduced at 100 µl per well for incubating at 4°C overnight. Rabbit anti-IgNAR pAb (GeneTex, GTX128445) diluted 1:3,000 in 3% BSA-PBS was added for incubation at room temperature for 2 h. Goat anti-rabbit IgG antibody HRP conjugate (Vector Laboratories, PI-1000-1) diluted 1:5,000 in 3% BSA-PBS was then added as a secondary antibody and then incubated at RM for 1 h. ELISA was developed with TMB substrate (Abcam, ab171522) and then read at 450 nm. Three shark individuals in each immunization program were involved in the above analyses.

### SDS-PAGE and Western Blot

For non-reducing 5% SDS-PAGE gel, 1 µl of plasma was heated at 65°C for 10 min in a SDS-loading buffer (50 mM Tris-Cl pH6.8, 2% SDS, 0.1% bromophenol blue, and 10% glycerol) without any reducing agents; for reducing 10% SDS-PAGE gel, 1 µl of plasma was boiled at 100°C for 5 min in the SDS-loading buffer with 100 mM DTT. The gels were run in a SDS-running buffer (3.03 g Tris base, 14.44 g glycine, and 1 g SDS in 100 ml of MilliQ-filtered H_2_O) at 120 V for 1–2 h.

For Coomassie Brilliant Blue (CBB) staining, the gel was soaked in 100 ml of a gel-fixing buffer (50% ethanol with 10% acetic acid in MilliQ water) for 1 h and then in 100 ml of a gel-washing buffer (50% methanol with 10% acetic acid in MilliQ water) at RM overnight with gentle agitation. Then the gel was stained in a CBB-staining buffer (0.1% CBB R350, 20% methanol, and 10% acetic acid in MilliQ water) at RM for 3–4 h. The gel was washed with the gel-washing buffer several times until clear bands appeared and then equilibrated in a storage buffer (5% acetic acid in MilliQ water) for 1 h before visualizing protein bands.

For Western blot, SDS-PAGE gels were blotted onto 0.45 µm PVDF membranes (Millipore) using the Mini Trans-blot system (Bio-Rad) at 4°C overnight. The membranes were then blocked with 5% skimmed milk-TBST at RM for 3 h. The blocked membranes were probed with rabbit anti-IgNAR pAb (GeneTex, GTX128445) diluted 1:3,000 in 5% skimmed milk-TBST at 4°C overnight, followed by goat anti-rabbit IgG antibody HRP conjugate (Vector Laboratories, PI-1000-1) diluted 1:5,000 in 5% skimmed milk-TBST. Similarly, the blocked membranes were probed with anti-IgW [V43] (Vertebrate Antibodies, #153309) or anti-IgM [Z69] (Vertebrate Antibodies, #153475), followed by horse anti-mouse IgG antibody HRP conjugate (Vector Laboratories, PI-2000-1). The membranes were washed in TBST four times prior to detection with the WesternBright Quantum kit (Advansta, K-12042-D10). For detecting vNARs and VHHs, mouse anti-His tag mAb HRP conjugate (Sino Biological, 105327-MM02T-H) was diluted 1:3,000 in 5% skimmed milk-PBS for membrane incubation.

Protein electrophoresis in agarose gels was performed to separate plasma proteins larger than 300 kDa. A 1.5% horizontal agarose gel was prepared in a resolving buffer (90 mM Tris base, 90 mM Boric acid in MilliQ water) and then electrophoresed in a running buffer (90 mM Tris base, 90 mM Boric acid, and 0.1% SDS in MilliQ water). The procedures for CBB staining and Western blot were the same as those for polyacrylamide gels. The protein bands on the gels or membranes were detected using an Azure c600 imaging system (Azure Biosystems).

### Germline IgNAR Gene Mining in *C. plagiosum*

The amino acid sequences of the V and C gene regions of IgNARs from different cartilaginous fish were retrieved in the NCBI and IMGT databases; meanwhile, the previously published IgNAR sequences with domain assigned (Smith et al., 2012a; Crouch et al., 2013b) were used as a reference for the position boundary of the V and C regions. The reference sequences of Chondrichthyes D and J genes were gathered from the IMGT database. All collected sequences were subjected to multiple sequence alignment to divide and extract V, D, J, or C genes. Subsequently, these nucleotide/amino acid sequences from different domains were used as BLAST queries against the *C. plagiosum* genome (Zhang et al., 2020a), following repeated filtration and detailed manual confirmation. To determine recombination signal sequence (RSS), several conserved heptamer and nonamer sequences were collected from previously released studies (Zhu et al., 2011; Sun et al., 2012; Carmona and Schatz, 2017), and an in-house Perl script was built to mine RSS in the potential IgNAR gene clusters of *C. plagiosum*. Two and three nucleotide mismatches were permitted to align heptamer and nonamer sequences to the genome, respectively. A detailed manual calibration was accomplished, and the RSS sequences of IgNAR genes were determined based on the results of the above integrated analysis. Finally, the structural configuration of seven IgNAR gene clusters in *C. plagiosum* was depicted with the detailed localizations of V-D-J and C genes and the structural features of RSS.

### Rapid Amplification of cDNA Ends and Phylogenetic Analysis

Total RNAs were extracted from whole blood cells of bamboo shark and then reverse transcribed to cDNA. The IgNAR gene-specific primer pairs were IgNAR_For_RACE (5ʹ-GATTACGCCAAGCTTCGAVTCAYTGACCATCAAYTG-3ʹ) and IgNAR_Re_RACE (5′-GATTACGCCAAGCTTTTYACAGTCASAMGGGTGCCG-3′). Both primers had a 15 bp extension (5ʹ, underlined in sequence) to facilitate In-Fusion cloning. The 5ʹ and 3ʹ cDNA fragments were generated using RACE with the SMARTer RACE 5′/3′ Kit (Clonetch Laboratories, 634859). The In-Fusion cloned 5ʹ- and 3ʹ-RACE products were sequenced and then assembled to obtain the full IgNAR sequences. IgNAR sequences from other cartilaginous fish were extracted from NCBI to construct a phylogenic tree using MEGA-X (version 10.1.8). The domain demarcation (Variable-V domain, Constant-C domains, Secretory-Sec tail, and Transmembrane-Tm tail) of IgNAR was performed as previously described (Greenberg et al., 1995; Feige et al., 2014). N-linked glycosylation sites of IgNAR were predicted by NetNGlyc 1.0 server (DTU Health Tech, Denmark). Basing from the amino acid sequences of IgNAR and the individual domains of IgNAR, we constructed phylogenetic trees using the minimum evolution method by MEGA-X. The number of bootstrap replications was set at 1,000. The trees generated were then visualized by the online tool iTOL (version 5.7).

### Analysis of Tissue-Specific IgNAR Expression

Different tissues, including brain, eye, gill, liver, spleen, pancreas, spiral valve, and blood, were collected from each of three adult and three infant *C. plagiosum* individuals, respectively. Total RNAs extracted from these tissues were sequenced with BGISEQ-500 platform using the PE150 strategy. The sequencing data were rRNA-removed using SOAP (version 2.21t) (Li et al., 2009) and filtered by SOAPnuke (version 1.5.6). Clean data were then aligned to the *C. plagiosum* genome (Zhang et al., 2020a) using HISAT2 (version 2.1.0) (Kim et al., 2015) and assembled using StringTie (version 1.0.4) (Pertea et al., 2015). All assemblies were merged by Cuffmerge (version 2.2.0). Reliable transcripts with potential protein-coding capacity (length > 200bp, exon > 2, FPKM > 1, coverage > 3 transcripts in any of the samples, and pFam E-value ≤ 0.001) were collected after filtering by Sixpack (version 6.6.0) and HMMER3 (version 3.1b1). Then the transcriptome from genome *de novo* annotation was integrated with the newly identified transcripts into a novel reference transcriptome exclusively for *C. plagiosum*. A pipeline for identifying gene expression levels based on the newly integrated reference transcriptome was implemented using RSEM (version 1.3.1) (Iqbal et al., 2012). The IgNAR gene clusters on the genome were used to identify IgNAR-specific mRNAs with an in-house Perl script, and the expression levels of IgNARs were determined basing from the RSEM results of the corresponding genes. Finally, the tissue differential expressions of the four IgNAR clusters (IgNAR1, IgNAR2, IgNAR5, and IgNAR6) represented as a normalized expression level based on the raw FPKM (fragments per kilobase of exons per million fragments mapped) were compared and presented using R scripts.

### NGS Library Construction of vNAR Repertoire

Total RNAs were extracted from PBMCs of bamboo sharks at well-defined time points during immunization by using the acid guanidinium thiocyanate–phenol–chloroform extraction method (Chomczynski and Sacchi, 2006). Complementary DNA (cDNA) was then synthesized with the SuperScript™ III First-Strand Synthesis System (Invitrogen, #18080051), following the product manual. vNAR DNAs were PCR amplified using the vNAR-specific primer pairs: vNAR_V (5ʹ-AGA​CCG​CTT​GGC​CTC​CGA​CTT​GGG​TTG​AAC​AAA​CAC​CGA​CA-3ʹ) and vNAR_J (5ʹ-ACA​TGG​CTA​CGA​TCC​GAC​TTA​ATC​CAT​TTG​CCC​TCT​GTT​CT-3ʹ). Next, the second-round PCR was conducted for DNA barcoding to differentiate each sample. Following this, DNA size selection and purification were performed with SPRIselect beads (Beckman Coulter, 23318). The ratio of PCR products and SPRIselect beads was 1:1 (v:v). Then the purified adaptor-ligated vNAR DNAs (300 ng in 48 μl TE buffer) were single-stranded by denaturation (heated at 95°C for 3 min and then immediately stored at 4°C for 2 min) and then circularized by T4 ligation (the above denatured DNA in 48 µl of TE buffer, 300 U T4 ligase, 0.83 µM split oligo primer, and 1 mM ATP in 12 µl of TA buffer) at 37°C for 30 min. Then the uncircularized vNAR DNA was removed by digestion (the above single-stranded circularized DNA in 60 µl buffer, 40 U Exonuclease I, and 65 U Exonuclease III in 1.4 µl of TA buffer) at 37°C for 30 min. Then the reaction was terminated with 7.5 µl of 0.5 M EDTA buffer. Following this, 1.3x AMPure XP beads (Beckman Coulter, A63881) were used to clean the reaction products, and then the purified single-stranded circularized DNA (sscDNA) was eluted in 20 µl of water. The final sscDNA concentration should be ≥1 ng/μl and quantified using the Qubit ssDNA Assay Kit (Invitrogen, Q10212) before sequencing.

### Comprehensive Bioinformatic Analysis for vNAR Immune Repertoire

The sscDNA representing the vNAR repertoire was sequenced using BGISEQ-500 platform with the SE400 model. An immune repertoire analysis was conducted based on the single-end 400 bp reads using IMonitor (version 1.4.1) (Zhang et al., 2015a). In brief, after sequencing, all reads were first filtered and trimmed using SOAPnuke (version 1.5.6) (Chen et al., 2018) to remove adapter sequence and reads with low qualities or excessively high N content. A new database of germline genes exclusively for *C. plagiosum* was built based on the sequences identified in the above germline gene mining process. Clean reads that met the filter criteria were aligned to the V and J germline sequences of bamboo shark IgNARs by BLAST (version 2.9.0) (Altschul et al., 1990) with specific parameters to accommodate the differences in the lengths of V/D/J segments. The high similarity of germline genes complicated accurate alignment. Thus, a secondary alignment process that combined global alignment and bootstrapping (base-by-base) extension strategies was implemented to identify the V/D/J genes accurately. On the basis of the determined V-J assignment, the DNA sequences were translated to protein to find the CDR3 region. Following this, we performed the statistical analysis on the V-J pairing and usage, unique CDR3 number, amino acid characteristics, high-frequency clone number, and clonotype overlapping. The diversity of CDR3 amino acids was evaluated based on the Shannon–Wiener index (Stewart et al., 1997) and the variability of amino acids as previously published tactics (Feng et al., 2018).

### Construction of vNAR-Phage Library

Total RNAs were extracted from PBMCs of bamboo sharks and then reverse transcribed to cDNA following the protocol of SuperScript III First-Strand Synthesis System (Invitrogen, 18080051). The vNAR DNAs were amplified by two steps of PCR. The primer pairs for the first-step PCR were as follows: NAR001 (5ʹ-GYGCAGAAACAATGAATATTTTCT-3ʹ) and NAR002 (5ʹ-GGATAGTAYCCGSTRATSAGACA-3ʹ). The primer pairs for the second-step PCR were as follows: vNAR_For (5ʹ-GAT​GTG​CAG​CTG​CAG​GAG​GGG​TTG​AAC​AAA​CAC​CGA​CA-3ʹ) and vNAR_Back (5ʹ-CTA​GTG​CGG​CCG​CAA​TCC​ATT​TGC​CCT​CTG​TTC​T-3ʹ). After two steps of PCR, the PCR products were performed with clean-up using the QIAEXII gel extraction kit (QIAGEN, 20051). The purified vNAR DNAs and the phagemid pMECS were double-digested by restriction endonucleases PstI-HF (NEB, R3140M) and NotI-HF (NEB, R3189M), respectively, at 37°C overnight. The digestion products were performed with clean-up using the QIAquick gel extraction kit (QIAGEN, 28706). The purified vNAR DNAs were ligated into the pMECS vector by T4 DNA ligation (1 μg vNAR DNA, 3 μg pMECS, and 10U T4 DNA ligase in 200 µl of ligation buffer) at 16°C overnight. The ligation reaction was used for transformation after heat inactivation at 70°C for 15 min. Electroporation was carried out in a 0.1 cm gap cuvette using a 1 µl ligation reaction in 25 µl of *E. coli* TG1 electrocompetent cells (Lucigen, ER2738). The TG1 cells were then plated on Amp-selective medium to generate a vNAR library of more than 10^7^ individual transformants. Following this procedure, the TG1 cells were collected for subsequent phage display and panning.

### Phage Display and Panning

TG1 cells bearing the phagemid library (∼10^10^ cells) were cultured in 2xTY/Amp-Glu medium (16 g tryptone, 10 g yeast extract, 5 g NaCl, 100 μg/ml Amp, and 2% D-glucose in 1 L of MilliQ water) at 37°C for 3 h. Then the cells were infected with M13K07 helper phages (NEB, N0315S) at a multiplicity of infection of 20 to produce a phage-displayed vNAR library. After an overnight culture, the amplified phage particles were precipitated using PEG/NaCl solution (20% polyethylene glycol 6,000, 2.5 M NaCl in MilliQ water) at 4°C for 1 h. About 1 × 10^11^ phage particles were incubated in each GFP-coated (or iRFP713-coated) well of MaxiSorp plate (BioLegend, 423501) for vNAR binding with GFP (or iRFP713) at RM for 2 h. The unbound phage particles were washed away with PBS/0.05% Tween, and the GFP-bound (or iRFP713-bound) phages were eluted for the consecutive rounds of panning using the same protocols mentioned above. Three to four rounds of panning were sufficient to enrich GFP-specific (or iRFP713-specific) phage particles.

### Phage ELISA

The phage ELISA was used to assess the enrichment of antigen-specific phage particles. GFP (or iRFP713) (100 ng) was coated per well at 4°C overnight and then blocked with 5% skimmed milk-PBS at RM for 3 h. The phage particles amplified after each round of panning were diluted into 2 × 10^10^ phages in 100 µl of 3% skimmed milk-PBS and then incubated in wells at RM for 2 h. Anti-M13 mAb HRP conjugate (Abcam, ab50370) diluted 1:3,000 in 5% skimmed milk-PBS was added and then incubated at RM for 1 h. The ELISA was developed with TMB substrate (Abcam, ab171522) and then read at 450 nm.

### Identification of Antigen-Specific vNARs

About 96–192 TG1 colonies randomly picked from LB-Amp agar plates were individually cultured in 1 ml of TB-Amp medium (1.15 g KH_2_PO_4_, 8.2 g K_2_HPO_4_∙3H_2_0, 6 g tryptone, 12 g yeast extract, 2 ml glycerol, and 100 μg/ml Amp in 0.5 L of MilliQ water) in each well of a 96-deep well plate. The same cells were placed on a reference master LB-Amp-Glu plate for temporary cell conservation. The 96-deep well plate was incubated at 37°C with shaking at 250 rpm for 3–5 h until OD_600_ reached 0.6. Then 1 µl of 1 M IPTG was added to induce the vNAR expression overnight at 37°C with shaking at 200 rpm. Next morning, the plate was centrifuged to pellet bacteria, and then TES-TES/4 buffers (TES: 0.2 M Tris-HCl pH 8.0, 0.5 mM EDTA, and 0.5M sucrose; TES/4: 1 volume TES buffer and 3 volumes MilliQ water) were used to lyse the cells as previously described (Pardon et al., 2014). Then the supernatant of the cell lysate was used to perform ELISA to identify GFP-specific (or iRFP713-specific) clones.

### Protein Expression and Purification

Two antigens, GFP and iRFP713, were used for bamboo shark immunization. Purified GFP (sequence from Addgene Plasmid #52107) was kindly provided by Dr. Qingxiang Sun at Sichuan University. The iRFP713 gene (sequence from Addgene Plasmid #31856) was cloned into pET32(a+) plasmid, with a N-terminal 6*His tag and a thrombin cleavage site. The plasmid was transformed into Shuffle T7 cells for protein expression under the 0.1mM IPTG induction. The protein expression was checked by Coomassie blue stained protein gel and Western blot. The protein was purified by immobilized metal affinity chromatography, followed by size exclusion chromatography.

TG1 cells bearing the vNAR or VHH expression plasmid were cultured in 0.5 L of TB-Amp medium (1.15 g KH_2_PO_4_, 8.2 g K_2_HPO_4_∙3H_2_0, 6 g tryptone, 12 g yeast extract, 2 ml glycerol, and 100 μg/ml Amp in 0.5 L of MilliQ water) at 16°C for overnight under 1 mM IPTG induction. For cell lysis, the cell pellet collected by centrifugation was first resuspended in 8 ml of TES buffer (0.2 M Tris-HCl pH 8.0, 0.5 mM EDTA, and 0.5 M sucrose) at 4°C for 6 h with rotation at 200 rpm and then mixed in 16 ml of TES/4 buffer (1 volume TES buffer and 3 volumes MilliQ water) at 4°C for 2 h with rotation at 200 rpm. After centrifugation, the supernatant was collected, and the pellet was subjected to a second cell lysis in the TES-TES/4 buffer. Then the periplasmic extracts were filtered through 0.22 µm syringe filters (Merck, SLGS033SB) and then added with 1 ml of IMAC nickel resin (Bio-Rad, 1560135) for affinity capture of His-tagged nanobodies. After an overnight gentle shaking at 4°C, the nickel resin was collected by gravity and washed with 30 ml of PBS by draining at gravity. The protein was eluted in the 5 ml of PBS-Imidazole buffer (150 mM imidazole in PBS). The imidazole can be removed by Amicon Ultra 3 kDa centrifugal filters (Merck, UFC900308), and the protein can be further purified using size exclusion chromatography. Protein purity was checked using the CBB-stained SDS-PAGE gel. The bivalent vNARs were cloned into the pMECS vector and then transformed into TG1 cells. The other procedures for bivalent vNARs expression and purification were the same as that of monovalent vNARs.

### EC50 Determination

For the EC50 determination of vNARs, 100 ng of GFP was coated per well and blocked with 5% skimmed milk-PBS. Tenfold serial dilutions of purified His-tagged vNARs (10^−3^ nM–10^4^ nM) were prepared in 5% skimmed milk-PBS and then incubated at RM for 2 h. Mouse anti-His tag mAb HRP conjugate (Sino Biological, 105327-MM02T-H) diluted 1:3,000 in 5% skimmed milk-PBS was then added for incubating at RM for 1 h. The ELISA was developed with TMB substrate (Abcam, ab171522) and then read at 450 nm.

### SPR for KD Determinations

The kinetics binding and dissociation between sdAb and antigen were monitored with SPR by Biacore T200 (Cytiva, United States). In brief, a 1-min pulse of the Ni solution (0.5 mM NiCl_2_ in water) was injected to saturate the NTA chip with nickel. Then his-tagged sdAbs (20 μg/ml in HBS-P buffer: 0.01 M HEPES pH 7.4, 0.15 M NaCl, and 0.005% v/v Surfactant P20) were captured on the flow cell of Sensor Chip NTA (Cytiva, 28994951). Then a serial of double-fold dilution of GFP (or iRFP713) was injected, and the sensorgrams were globally fitted with a floating R_max_ using the built-in evaluation software. The binding and dissociation times were set at 120 and 300 s, respectively. At last, the regeneration solution (350 mM EDTA) was used to remove nickel and any chelated molecules on the chip surface. The binding affinity (KD) was calculated as KD (nM) = Kd (1/s)/ Ka (1/Ms), where Kd is the dissociation constant and Ka is the association constant. Three replicates of SPR analysis were conducted for each sdAb.

### Overlapping/Non-Overlapping Epitope Prediction

For the overlapping/non-overlapping epitope prediction of vNARs and VHHs, one His-tagged sdAb (named as sdAb1) was coated with 40 ng per well and then blocked with 5% skimmed milk-PBS. Then 400 ng of GFP was incubated per well for sdAb binding. The other His-tagged sdAbs (named as sdAb2) were then individually incubated with 400 ng per well for competitive epitope binding. The sdAb1 was added either as a control. Mouse anti-His tag mAb HRP conjugate (Sino Biological, 105327-MM02T-H) was then added for binding with His-tagged sdAbs. The ELISA was developed with TMB substrate (Abcam, ab171522) and then read at 450 nm. The non-overlapping epitopes existing between sdAb1 and sdAb2 were identified if the well added with sdAb2 has higher absorbance values than the control.

### Validation of Intrabody Expression

For the validation of intrabody expression, a 12-well plate of 293T cells was transfected with plasmids bearing vNAR and VHH insertion by Lipofectamine 3000 (Invitrogen, L3000015) in accordance with the product protocol. Transfected 293T cells were cultured at 37°C, 5% CO_2_ for 48 h. Cell lysates were prepared by incubation with RIPA buffer containing protease inhibitor cocktail (Bimake, B14001). After incubation on ice for 30 min, cell lysates were centrifuged for 10 min at 10,000 rpm, and supernatants were boiled with the SDS-loading buffer for 5 min. Then total protein samples were separated by SDS-PAGE gel and then transferred onto PVDF membranes (Millipore). The membranes were blocked in TBST containing 5% skimmed milk and incubated in primary antibodies diluted in blocking buffer at 4°C for overnight. Primary antibodies for immunodetection were sourced as follows: Flag antibody (CST, 2368S) and β-actin antibody (ABBKINE, A01010).

### Immunoprecipitation

293T cells expressing GFP in 10 cm plate were transfected with plasmids bearing vNAR and VHH insertion by Lipofectamine 3000 (Invitrogen, L3000015) in accordance with the product protocol. Transfected 293T cells were cultured at 37°C, 5% CO_2_ for 48 h. Cells were lysed in 500 µl of lysis buffer (1% NP40, 25 mM Tris pH 7.5, 150 mM NaCl, and protease inhibitor cocktail (Bimake, B14001). After incubation on ice for 30 min, cell lysates were centrifuged for 10 min at 10,000 rpm, and supernatants were diluted to 500 µl of binding buffer (25 mM Tris pH7.5 and 150 mM NaCl). A 4 μg Flag antibody (Sigma, F3165) was incubated with 50 µl of magnetic protein G beads (Bio-Rad, 1614023) for 30 min at RM, added with diluted cell lysates, and then further incubated at 4°C overnight. The beads were washed four times with the washing buffer (0.5% NP40, 25 mM Tris pH 7.5, and 300 mM NaCl) before analysis. Beads were boiled with SDS loading buffer for 5 min, and then the supernatants were loaded into SDS-PAGE gel. Primary antibodies for immunodetection were sourced as follows: Flag antibody (CST, 2368S) and GFP antibody (CST, 2956S).

### Statistical Analysis

The analyses were conducted with GraphPad Prism 8 software. Comparisons between groups were performed using two-tailed t tests, and statistical significance was considered at *p* < 0.05. The Pearson correlation coefficient was used to assess the similarity between groups.

## Results

### Comprehensive Characterization of IgNAR

**FIGURE 1:**
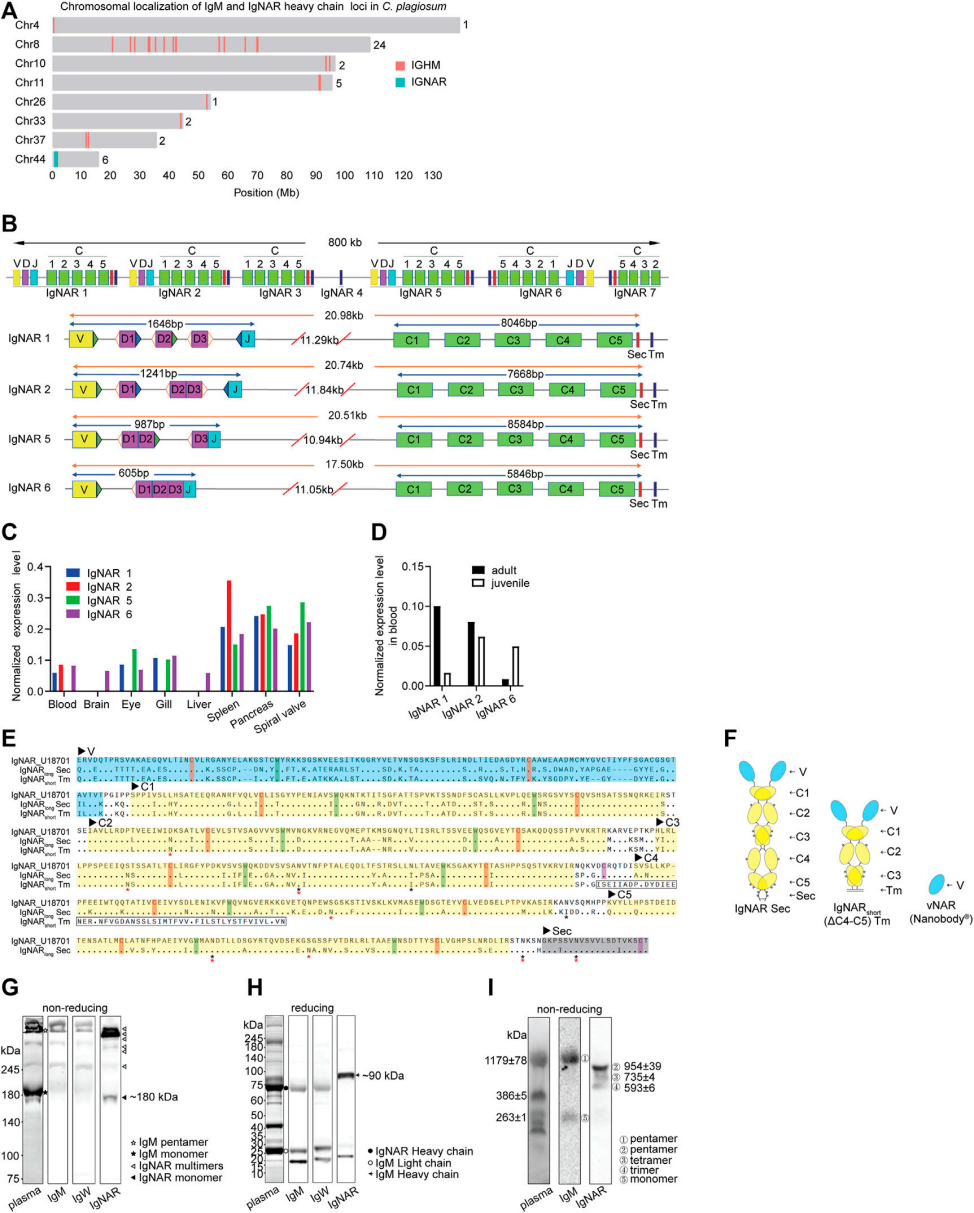
Characterization of IgNAR in bamboo sharks. **(A)** Chromosomal localizations of IgM loci and IgNAR loci. Numbers behind the chromosomes represent the numbers of loci in the respective chromosome. **(B)** Germline configurations of seven IgNAR clusters. The position and orientation of recombination signal sequences (RSS) are present beside the Variable (V), Diversity (D), and Joining (J) gene segments, whereas hollow triangles represent 12-bp spacer RSS, blue solid triangles represent 23-bp spacer RSS, and green solid triangles represent 22-bp spacer RSS. “C,” constant domains; “Sec,” secretory tail; and “Tm,” transmembrane tail. **(C, D)** Expression level of IgNAR mRNA in different tissues **(C)** and age **(D)** revealed by multi-tissue transcriptome. **(E)** Protein sequence alignment of two IgNAR isoforms from bamboo shark with *Ginglymostoma cirratum* (Gc) IgNAR (Genbank U18701). Variable (V, blue shading) and constant (C, yellow shading) domains, and Sec (grey shading) and Tm (boxed) tails are indicated. Conserved cysteines for intradomain disulfide bonds (red) and for inter-domain disulfide bonds (purple) and tryptophan (blue) are highlighted. Asterisks indicate potential N-linked glycosylation sites (Red for IgNAR_long_ Sec and IgNAR_short_ Tm, and Black for U18701). **(F)** Schematic representation of IgNAR_long_ Sec, IgNAR_short_ Tm and vNAR. **(G, H)** Three Ig isotypes (IgM, IgW, and IgNAR) in plasma were analyzed by non-reducing **(G)** and reducing **(H)** SDS-PAGE and WB. The blocked membranes were probed with rabbit anti-IgNAR pAb, anti-IgW or anti-IgM, followed by secondary antibody HRP conjugate before detection. IgM monomer, 200–220 kDa; IgM pentamer, 1,000–1,200 kDa; IgNAR monomer, 180–200 kDa; IgM heavy chain, ∼75 kDa; IgM light chain, ∼25 kDa; and IgNAR heavy chain, ∼90 kDa. **(I)** Separation of IgM multimers and IgNAR multimers. Circled numbers indicate ①-IgM pentamer (1179 ± 78 kDa), ②-IgNAR pentamer (954 ± 39 kDa), ③-IgNAR tetramer (735 ± 4 kDa), ④-IgNAR trimer (593 ± 6 kDa), and ⑤-IgM monomer (263 ± 1 kDa).

We discovered 37 IgM clusters distributed on seven chromosomes (Chr) and seven IgNAR clusters located near one end of Chr44 in the white-spotted bamboo shark genome (Figure 1A) (Zhang et al., 2020a). The numbers of IgNAR clusters in cartilaginous fish are mostly within 10, much lower than that of IgM clusters (Supplementary Figure S1A). Seven IgNAR clusters occupied a range of 800 kb on Chr44 (position in Mb: 0.56–1.36) and were spatially distant with each other. Four (IgNAR 1, IgNAR 2, IgNAR 5, and IgNAR 6) of seven IgNAR clusters had the complete IgNAR structure comprising one variable (V), three diversity (D), one joining (J), and five constant (C) gene segments, followed by one secretory tail (Sec) and one transmembrane tail (Tm); the other three were incomplete (Figure 1B). Moreover, pre-joined D gene segments existed in clusters IgNAR2, IgNAR5, and IgNAR6. Intriguingly, a conserved cysteine existed at the Sec carboxyl terminus of all three shark Ig isotypes and human IgA and IgM, which is critical for multimerization (Supplementary Figure S1B) (Smith et al., 2012b; Castro et al., 2013; Mashoof and Criscitiello, 2016). Transcriptomic analysis revealed that IgNARs were expressed in spiral valve, pancreas, and spleen (Figure 1C). IgNAR1 was preferentially expressed in adult sharks, whereas IgNAR6 was in juveniles (Figure 1D); IgNAR2 was expressed in both sharks.

Result of rapid amplification of cDNA ends (RACE) from blood revealed two IgNAR isoforms from the IgNAR1 cluster: IgNAR_long_ Sec form (V-C1-C2-C3-C4-C5) and IgNAR_short_ Tm form (V-C1-C2-C3) (Figures 1E,F). As aligned with nurse shark IgNAR, these IgNARs had similar constant domain sequences, conserved cysteines forming an intradomain or interchain disulfide bridge, conserved tryptophans, and potential N-linked glycosylation sites. Compared with other species, IgNARs in white-spotted bamboo shark were similar to those in species from the same order, that is, Orectolobiformes, including brownbanded bamboo shark, nurse shark, and spotted wobbegong (Supplementary Figure S1C). We discovered that bamboo shark possessed multiple secretory Ig multimers in plasma, including IgM pentamer, IgW pentamer and dimer (data not shown), and IgNAR trimer, tetramer, and pentamer. Our finding was based on the different molecular weights of shark Igs and the protein-specific antibodies for shark Igs (Figures 1G–I and Supplementary Figures S1D,E). In addition, the blots clearly indicated that IgNAR multimers, especially IgNAR pentamer, were the majority of secretory IgNAR isoforms in plasma.

### Humoral Immune Responses Upon Immunization

**FIGURE 2:**
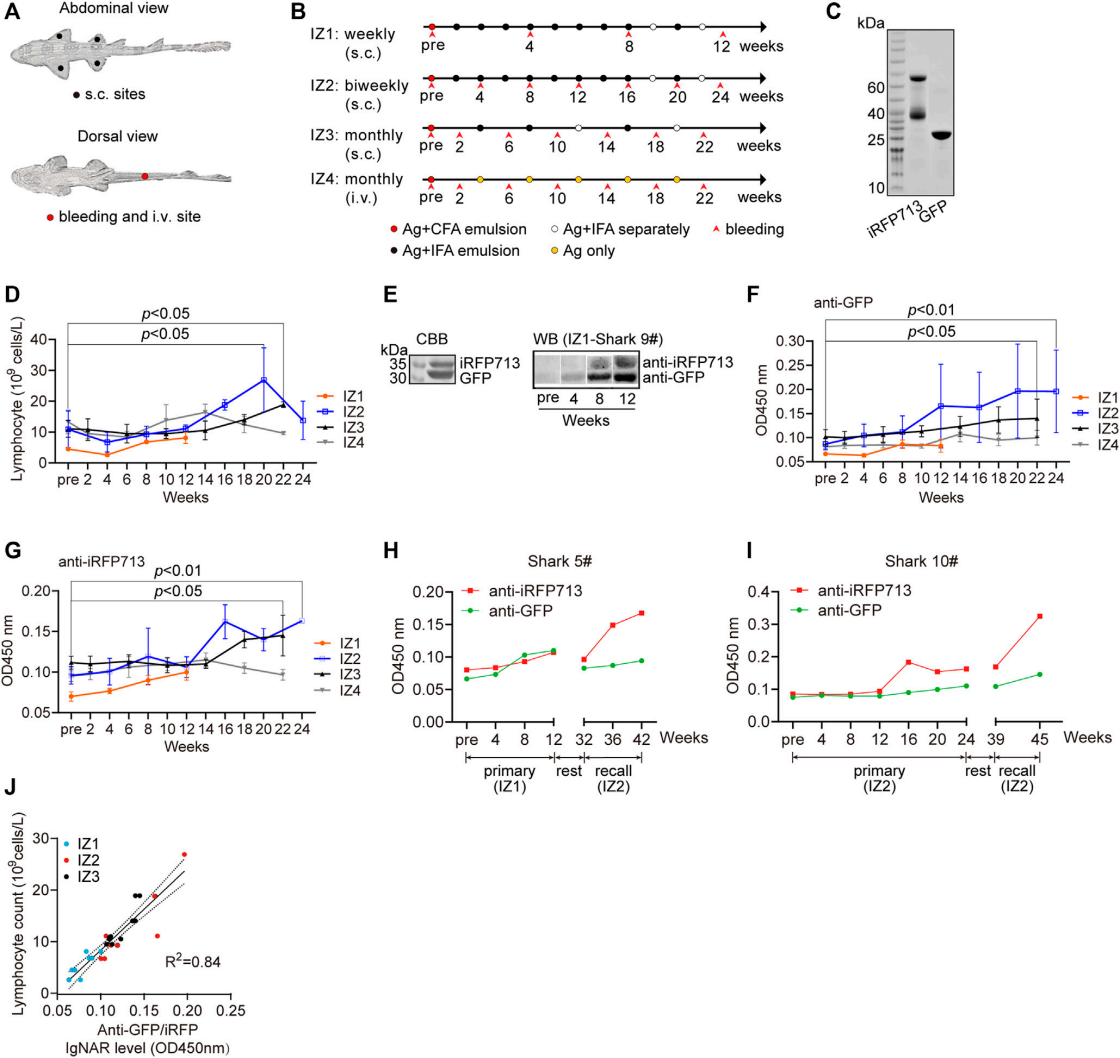
Immune responses of bamboo sharks to immunization. **(A, B)** The injection and bleeding sites **(A)** and the immunization schedules of four immunization programs **(B)**. s.c., subcutaneous; i.v., intravenous; Ag, antigen mixture; CFA, complete Freund’s adjuvant; and IFA, incomplete Freund’s adjuvant. **(C)** SDS-PAGE of two purified antigens, GFP and iRFP713. **(D)** Lymphocyte counts increased during immunization (*n* = 3). Statistical significance for the last post-immunization vs. the pre-immunization is indicated by asterisks (*p < 0.05). **(E)** Identification of antigen-specific IgNARs in plasma. **(F, G)** Antigen-specific IgNAR level in plasma increased during immunization (*n* = 3). Statistical significance for the post-immunization vs. the pre-immunization is indicated by asterisks (*p < 0.05, **p < 0.01). **(H, I)** Two sharks with extended immunization showed an enhanced IgNAR level. **(J)** IgNAR level were correlated with lymphocyte counts (*n* = 29).

To stimulate the effective immunity of bamboo shark upon immunization, we considered several immunization parameters, including the delivery route and injection sites (Figure 2A), the number of and time interval between injections, and the combination way of Freund’s adjuvants and antigens. Hence, we conducted four immunization programs with antigens GFP and iRFP713 (Filonov et al., 2011) (Figures 2B,C). Some sharks experienced more than 30 times of injection with five antigens in 3 years, and some sharks laid eggs and produced healthy sperms during the raising in small aquarium, indicating the potential for artificial reproduction (Supplementary Figures S2A–F). The potential side effects of injections were rare, and the hematologic values of immunized sharks were within normal limits under our raising and immunization strategies (Supplementary Figures S2G–H). These results demonstrate that bamboo shark is a robust shark species, readily adaptive to small water body, tagging, and repeated immunizations.

The IZ2 program had the highest increase in lymphocyte and white blood cells (WBC) counts during immunization progression (Figure 2D and Supplementary Figure S2I). The two counts for IZ1 were relatively low, but the antigen specific IgNAR level in plasma still increased (Figure 2E). The fluctuation of immune cell counts for IZ4 suggested an inapparent immune response. All immunization programs exhibited an increasing tendency in antigen specific IgNAR levels, indicative of the effective immunogenicity and efficacy raised by repeat immunizations (Figures 2F,G). According to the previous reports (Dooley, 2003; Dooley and Flajnik, 2005), the growth rate of antibody titer in sharks upon vaccination is much lower than that observed in mammals. Intriguingly, we observed a memory response of IgNAR because the anti-iRFP713 IgNAR recall response was more rapid and stronger than the primary response (Figures 2H,I). Moreover, a strong positive linear correlation was found between WBC or lymphocyte counts and antigen specific IgNAR level (Figure 2J and Supplementary Figure S2J). Together, we demonstrate that bamboo shark possesses humoral IgNAR immune response.

### Development of vNAR Immune Repertoire During Immunization

**FIGURE 3:**
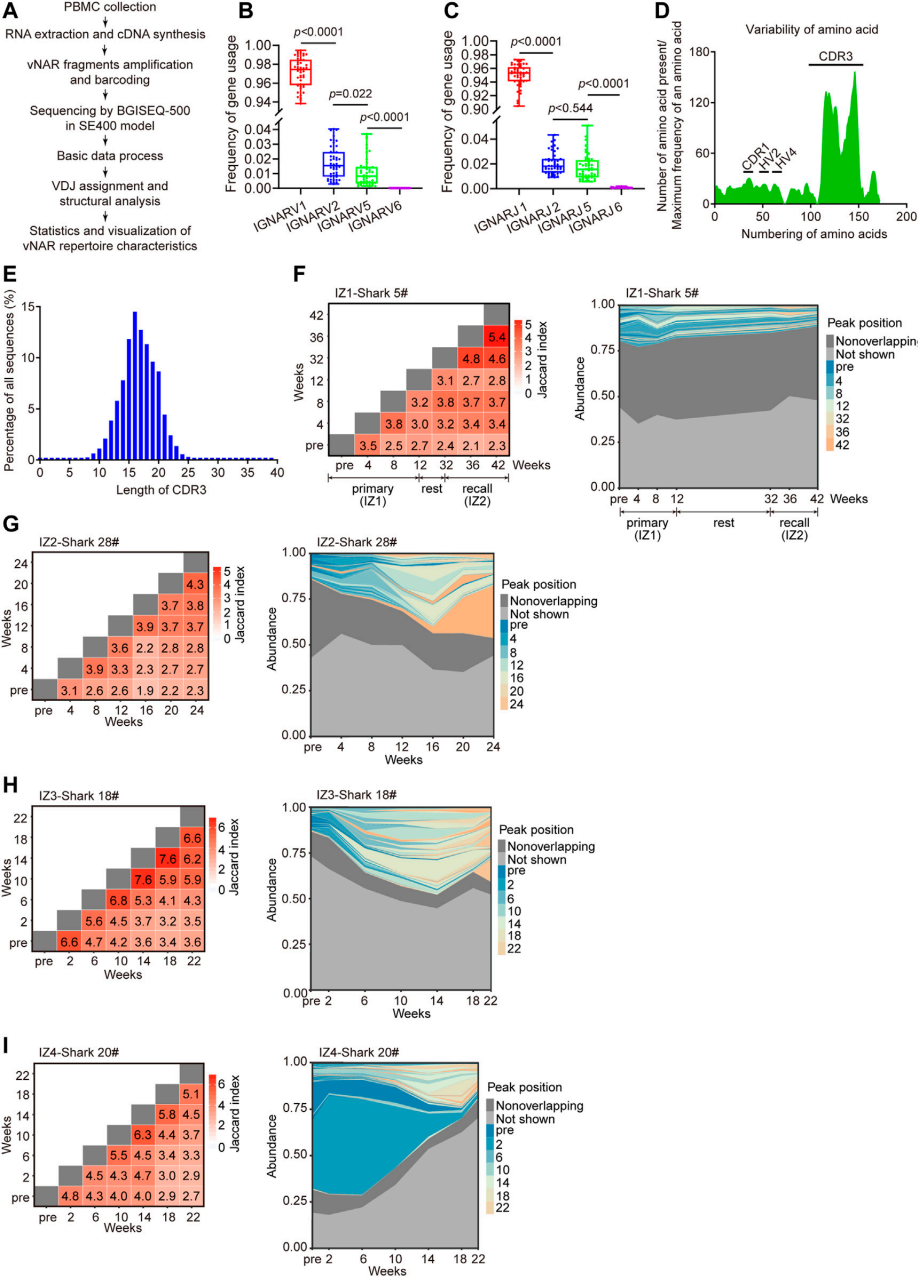
Development of vNAR immune repertoire of bamboo sharks during immunization. **(A)** Schematic for the characterization of vNAR immune repertoire constructed from immunized sharks. **(B, C)** Usage frequency of V/J genes from four IgNAR clusters. Statistical significance is indicated by the *p* value. **(D, E)** Variability of amino acid sequences **(D)** and CDR3 lengths **(E)** of vNARs (*n* = 3,00,000). CDR, complementarity determining region; HV, hypervariable region. **(F, I)** Clonotype overlap and clonotype tracking of vNAR immune repertoire of bamboo sharks during immunization. Peak positions represent the abundance profiles of top 100 clonotypes (colored), non-overlapping clonotypes (dark gray), and remaining (Not shown) clonotypes (light gray). Value in each tile of heatmap represents the clonotype overlap rate between two time points as shown on the two axes.

High-throughput sequencing of immune repertoire was conducted to display the development of vNAR immune repertoire in response to immunization (Figure 3A). More than 94 million of vNAR sequences were obtained from six immunized bamboo sharks (Supplementary Table S1). The ratio of unique vNAR sequences in one time-point for each individual was ranging from 63.6 to 95.3%. The IGNARV1-IGNARJ1 pairing usage from the IgNAR1 cluster (96%) contributed the most to the IgNAR immune repertoire in blood (Figures 3B,C). Few changes in the V/J gene usage profile were observed in all sharks during the immunization progression (Supplementary Figure S3A). The highest variabilities in the composition and length of amino acids (AA) were distributed at the complementarity determining region 3 (CDR3) region, which gave the largest contribution to vNAR binding diversity (Figures 3D,E). The CDR3 length of vNAR was broader than that of human VH (ranges from 4 to 36 amino acids) (Rock et al., 1994).

The immune repertoire sequencing provides us a predictive indicator of immunization efficacy, complementary to our serological measurements. Upon immunization, different immunization programs have different landscapes of vNAR immune repertoire except for the unanimous V-J gene usage frequency. Two immunization programs (IZ2 and IZ3) evidently skewed the vNAR immune repertoire toward the high-frequency vNAR clones, represented as the overall decreased unique CDR3 numbers, and the gradually decreased diversity of CDR3 AA (Shannon index) and the cumulative frequency of top 100 clones (Supplementary Figure S3B). However, the two other programs (IZ1 and IZ4) displayed the opposite results on these points because of their low-level immune responses (Supplementary Figure S3C). The low-frequency clones at initial immunization expanded into high-frequency clones upon repeated immunization (Figures 3F–I). In summary, these data indicate that the effective antigen driven IgNAR immune responses in bamboo sharks can be stimulated by repeated immunizations.

### Identification of High-Affinity vNARs From Immunized Library

**FIGURE 4:**
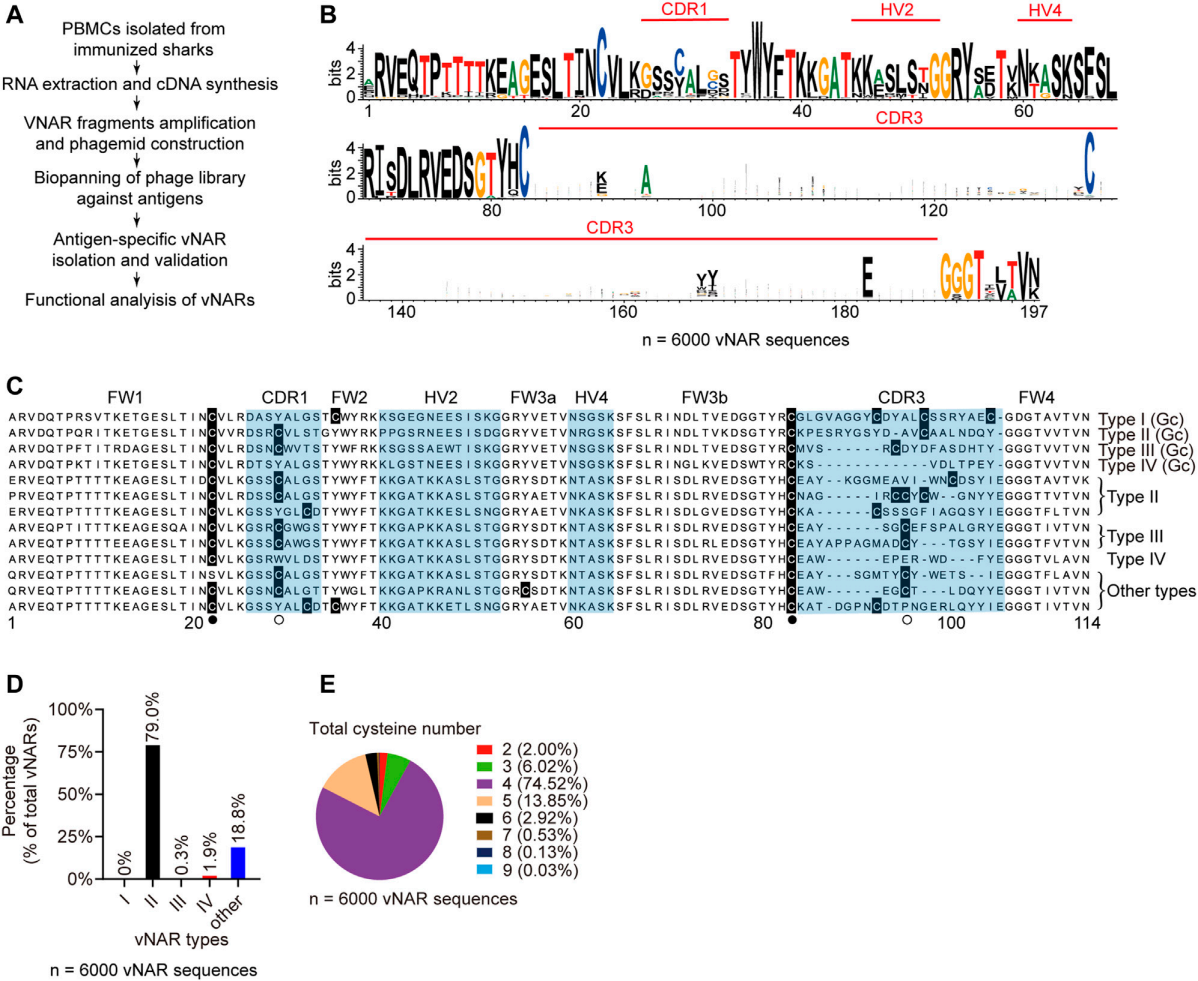
Characteristics of vNAR sequences from immunized library. **(A)** Schematic for vNAR phage library construction from immunized sharks. **(B)** Amino acid variability for vNAR sequences (*N* = 6,000). **(C, D)** Sequence alignment **(C)** and percentages **(D)** of different vNAR types in the library. **(E)** Percentages of vNARs with different cysteine numbers.

Then we isolated high-affinity antigen-specific vNARs from immunized sharks by phage display (Figure 4A). The vNAR-phage library, constructed from eight immunized sharks, contained 4.67 × 10^8^ individual transformants (Supplementary Figure S4A). The 339.5 million full-length vNAR sequences from the library revealed that 83.8% of vNARs were unique in sequence (Supplementary Table S2). The enormous sequence diversity in CDR3 resulted in several gaps in the alignment, showing a wider sequence region in the sequence logos (Figure 4B). Two canonical cysteines (Zielonka et al., 2015) for all vNAR types were located at FR1 and FR3b (Figure 4C). Two frequently used non-canonical cysteines were positioned at CDR1 and CDR3, and a highly conserved tryptophan residue in FR2 was positioned adjacent to the disulfide bond. Approximately 79.0% of total vNARs were the classical type II (Figure 4D). Some new types (18.8%) did not fit in any of the four known types (type I-IV). Type IV accounted for 1.9%. Type III produced by young sharks (<1-year-old) was fewer (Diaz et al., 2002). Furthermore, the majority (74.5%) of vNARs had four cysteines from type II (Figure 4E) which is similar to nurse shark (Feng et al., 2019). These data indicate that our bamboo shark vNAR library had high diversity in vNAR sequences and distinct features in vNAR type profile.

**FIGURE 5:**
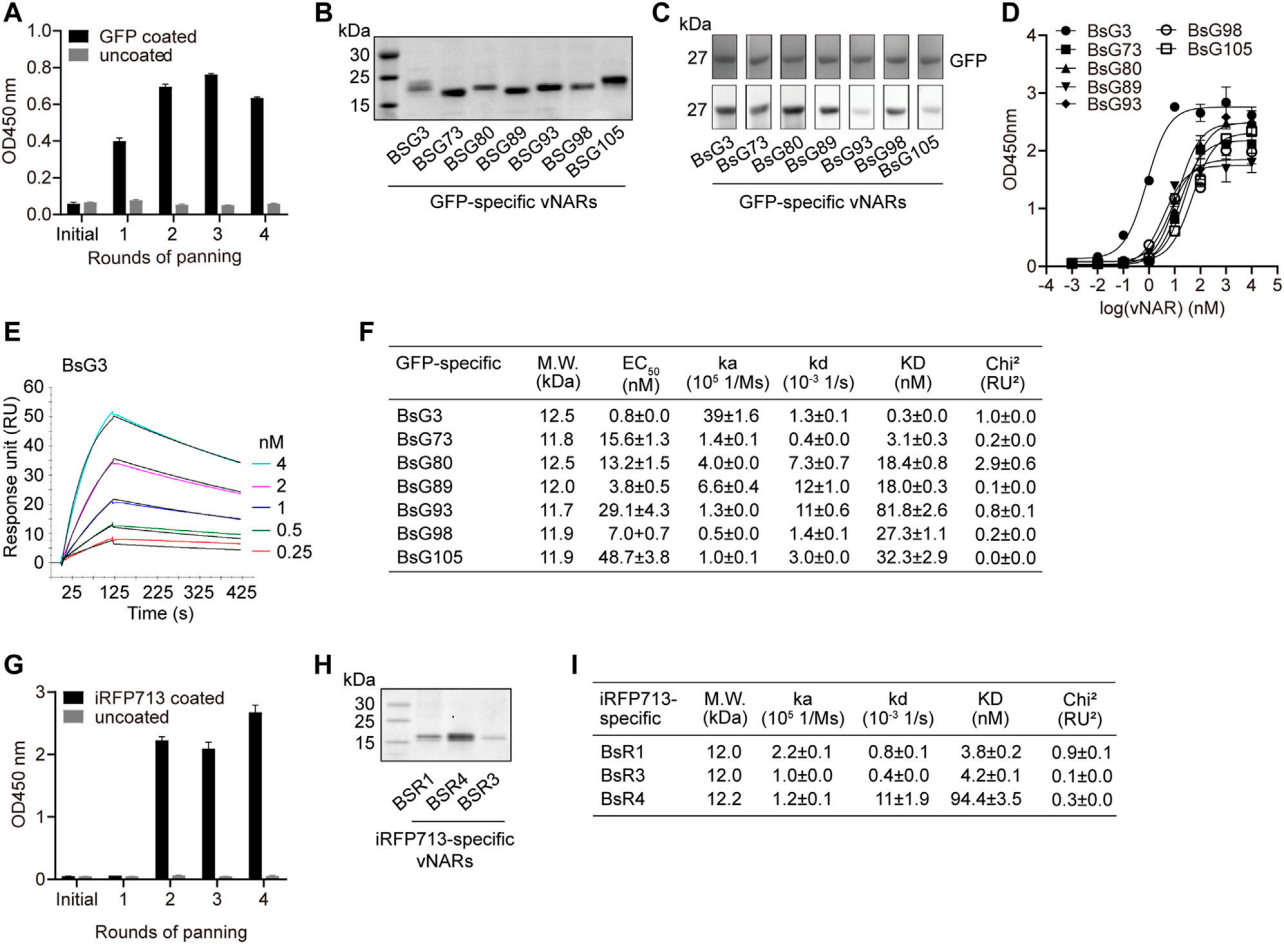
Identification of high-affinity GFP-specific and iRFP713-specific vNARs from immunized library. **(A)** Enrichment of GFP-specific vNARs during panning. **(B)** SDS-PAGE of seven purified anti-GFP vNARs. **(C)** Functional validation of vNARs. **(D)** EC_50_ of vNARs against GFP. **(E)** SPR sensorgram of BsG3 binding with GFP. **(F)** Binding affinities and EC_50_ of seven vNARs against GFP. The values represent mean ± standard error. **(G)** Enrichment of iRFP713-specific vNARs during panning. **(H)** SDS-PAGE of three purified anti-iRFP713 vNARs. **(I)** Binding affinities of three vNARs against iRFP713. The values represent mean ± standard error.

Seven GFP-specific vNARs were retrieved by four rounds of biopanning (Figures 5A,B and Supplementary Figure S4B). They were type II vNARs and recognized denatured GFP (Figure 5C). The ELISA EC50 value of each vNAR to GFP (Figure 5D) was consistent with its binding affinity (KD) measured by surface plasmon resonance (SPR) (Figures 5E,F and Supplementary Figures S4C–H). Five vNAR candidates originated from the IZ2 group as revealed by phylogenetic analysis (Supplementary Figure S4I). BsG3 with 0.3 nM binding affinity was originated from shark 28# in IZ2 group, which showed the highest GFP-specific IgNAR level. Moreover, three iRFP713-specific vNARs with nM binding affinities were isolated from the immunized library (Figures 5G–I and Supplementary Figures S4J–L). These data demonstrate that high-affinity vNARs can be generated from immunized bamboo sharks.

### Engineering of vNAR

**FIGURE 6:**
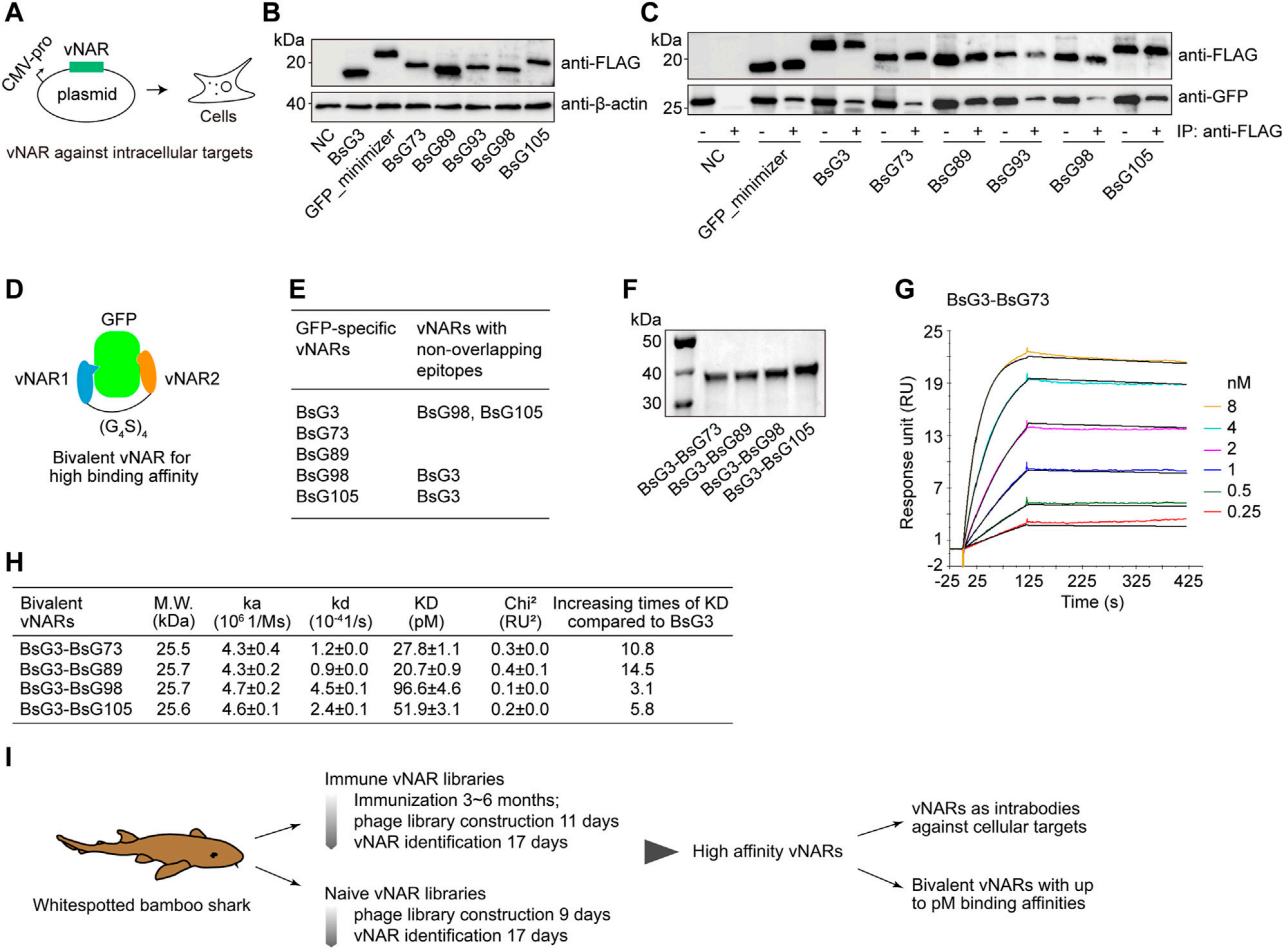
Validation of GFP-specific vNARs as intrabodies and bivalent constructs. **(A)** Schematic for vNARs as intrabodies in human cells. **(B)** Expression of vNARs in HEK293T cells. **(C)** Native GFP-vNAR complexes were pulled down from cell lysates. **(D)** Design of bivalent vNARs. **(E)** Mapping of binding epitopes. **(F)** SDS-PAGE of purified bivalent vNARs. **(G)** SPR sensorgram of BsG3-BsG98 binding with GFP. **(H)** Binding affinities of four bivalent vNARs to GFP. The values represent mean ± standard error. **(I)** Schematic for the identification of high-affinity binders from bamboo shark vNAR library and their applications.

To prove vNAR to be functional in cells (Figure 6A), vNARs were expressed in 293T cells together with GFP (Figure 6B). These vNARs bound to GFP revealed by the vNAR pull down experiment (Figure 6C). This indicates that vNARs are functional in mammalian cells. To improve vNAR affinity, the biparatopic vNARs with an internal (G_4_S)_4_ linker were constructed based on epitope mapping result; in specific, BsG3 recognized an epitope on GFP different from those of BsG98 and BsG105 (Figures 6D–F and Supplementary Figures S5A–L). The SPR analysis showed all bivalent vNARs reached a picomolar binding affinity with GFP representing the highest known GFP antibodies (Figures 6G,H and Supplementary Figures S5M–P). In summary, our data demonstrate that bamboo shark vNARs have versatile potentials in high-affinity vNAR discovery and vNAR multimerization to reach a picomolar affinity and function as intrabodies to modulate antigens in cells (Figure 6I).

## Discussion

We demonstrate bamboo shark as a promising small shark model for affordable high-affinity sdAb production due to several advantages. First, bamboo sharks are small demersal sedentary fish. The farming and handling of bamboo sharks are simple and cheap. Nurse sharks are the most used shark species for immunization for vNAR production (Dooley, 2003; Dooley et al., 2006; Steven et al., 2017). However, they are approximately 2–3 m long and require large and expensive culture devices. Second, bamboo sharks are harmless for operators and not endangered. Third, bamboo sharks have a short reproduction period, less than 5 years for both genders. We can raise many sharks for immunization through artificial breeding. Comparably, nurse sharks require long years (♀15–20, ♂10–15) to be mature for reproduction. In addition, we found age-related IgNAR expression profile differences in bamboo shark, which is similar to nurse sharks (Roux et al., 1998). Adult sharks should be immunized for vNAR discovery because adults have mature IgNAR repertoire. Fourth, bamboo shark is the first shark species with the complete structural configuration of all IgNAR clusters in chromosome. We could monitor the changes in antibody immune repertoire during immunization to develop enhanced methods for antibody identification. Fifth, the proper immunization strategies we established for bamboo sharks offer guarantee to build effective immunized vNAR libraries, resulting in a high likelihood for high-affinity vNAR isolation. Many researchers adopted monthly intravenous administration for shark immunization (Dooley and Flajnik, 2005; Flajnik et al., 2009; Crouch et al., 2013c). However, we found that the intravenous route is less effective in antigen stimulation than the subcutaneous route, which is simpler in operation and less harmful for sharks. In fact, intravenous injection is seldom used in animal vaccination because of its relatively low immune response and the risk of allergic reaction and toxicity (Zhang et al., 2015b). In addition, biweekly injection interval is better than monthly injection interval for efficient humoral immune response (e.g., 2-month saving in this study). Sixth, high-affinity and specificity vNAR binders can be generated from immunized bamboo shark library. Finally, bamboo shark vNARs can function as intrabodies in mammalian cells and have a simple multimerization strategy to reach high affinity. Our GFP-specific bivalent vNARs reach a picomolar affinity that is much higher than the bivalent VHH equivalents (Fridy et al., 2014; Zhang et al., 2020b) and recognize unique conformational epitopes.

Our data present the effective IgNAR immune response to antigen stimulation in bamboo sharks. Bamboo sharks are the third reported shark species possessing IgNAR multimers (the other two: spiny dogfish (Smith et al., 2012b) and small spotted cat shark (Crouch et al., 2013a)), whereas nurse sharks do not. Intriguingly, cartilaginous fish serum typically contains a higher level of multimeric forms than the monomeric form of the same Ig isotype, a feature other vertebrates lack (Dooley and Flajnik, 2005). Whether or not these Ig multimers have selected functional roles compared with their corresponding monomers is unknown. IgM pentamer is the first-line T-independent defense without involving antigen-driven humoral immunity (Dooley and Flajnik, 2005). Whether or not IgNAR multimers also have the same mechanism remains unclear. In bamboo shark, the Sec and Tm tails are spatially close in germline following the C5 region in each IgNAR cluster and share the same sets of V and C genes; monomeric and multimeric forms also share the same IgNAR cluster. Thus, how multimerization occurs and develops remains to be addressed in the future. Moreover, whether or not J chain is required for IgNAR multimer formation is unknown, although the secretory tail of IgNAR carries the necessary cysteine and N-linked glycosylation site. Interestingly, IgNAR_short_ Tm form lacks C4 and C5 domains, while a newly discovered bamboo shark IgNAR_short_ Sec form is devoid of C2 and C3 domains (Zhang et al., 2020c). This difference might be ascribed to the alternative splicing of IgNAR mRNAs. However, the functional roles of these minority isoforms of IgNAR remain to be determined.

Taken together, our comprehensive analysis on bamboo shark IgNAR and vNAR are expected to facilitate the study of IgNAR immune repertoire in other shark species. Moreover, our research pioneered bamboo shark as a promising shark model for affordable high-affinity vNAR sdAb discovery and development as immunoreagents and diagnostic reagents.

## Data Availability

The datasets presented in this study can be found in online repositories. The names of the repository/repositories and accession number(s) can be found in the article/Supplementary Material. The immune repertoire sequencing data were deposited in the CNSA (https://db.cngb.org/cnsa/) with the accession codes CNP0001746.
