# Supplementary material for: Bamboo Shark as a Small Animal Model for Single Domain Antibody Production
Source: Front Bioeng Biotechnol. 2021 Dec 8;9:792111. doi: 10.3389/fbioe.2021.792111 (PMC8692893; doi:10.3389/fbioe.2021.792111)

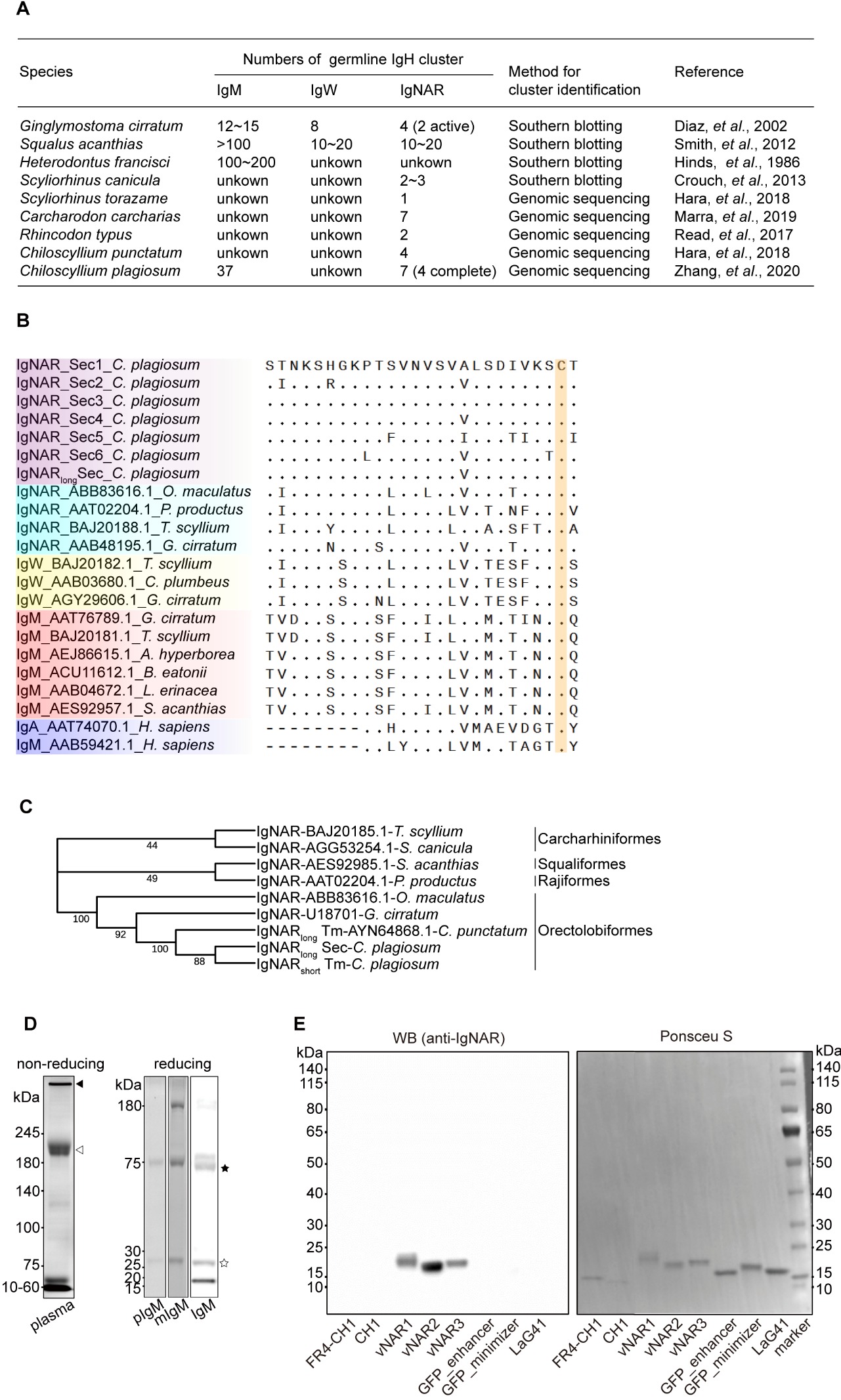


**Fig. S1. Sequence characterization of IgNARs and identification of secretory IgMs and IgNARs.** (**A**) Numbers of germline IgH clusters in different shark species. “2 active” means only two IgNAR clusters were expressed in adult *G. cirratum.* “4 complete” means four IgNAR clusters have the complete IgNAR structure (1V-3D-1J-5C). Diaz et al., 2002 (10); Smith et al., 2012 (28); Hinds et al., 1986 (61); Crouch et al., 2013 (20); Hara et al., 2018 (26); Marra et al., 2019 (62); Read et al., 2017 (63); Zhang et al., 2020 (25). (**B**) Amino acid sequence alignment of secretory tails of three immunoglobulin isotypes in different Chondrichthyes. NCBI accession number and shark/ray species were included in each sequence name. A conserved cysteine was located at the secretory carboxyl-terminus of immunoglobulins. (**C**) Phylogenetic analysis of full-length IgNARs in different Chondrichthyes. The order name of each organism was given beside the tree. (**D**) Identification of two IgM isoforms and their heavy and light chains by Bis-Tris SDS-PAGE. The IgM pentamer and monomer in gel slices were respectively resolved by reducing SDS-PAGE into heavy and light chains, and then recognized by anti-IgM mAb. This result confirmed the correct identification of IgM. The solid star represents the IgM heavy chain, and the hollow star the IgM light chain. The IgM pentamer (solid triangle) and IgM monomer (hollow triangle) are indicated. (**E**) Specificity validation of anti-IgNAR antibody. The result shown that the anti-IgNAR antibody recognized vNAR rather than the CH1 domain. Fr4-CH1, CH1, and three vNARs were generated from bamboo shark IgNAR. GFP_enhancer (64), GFP_minimizer (64), and LaG41 (37) were VHHs.


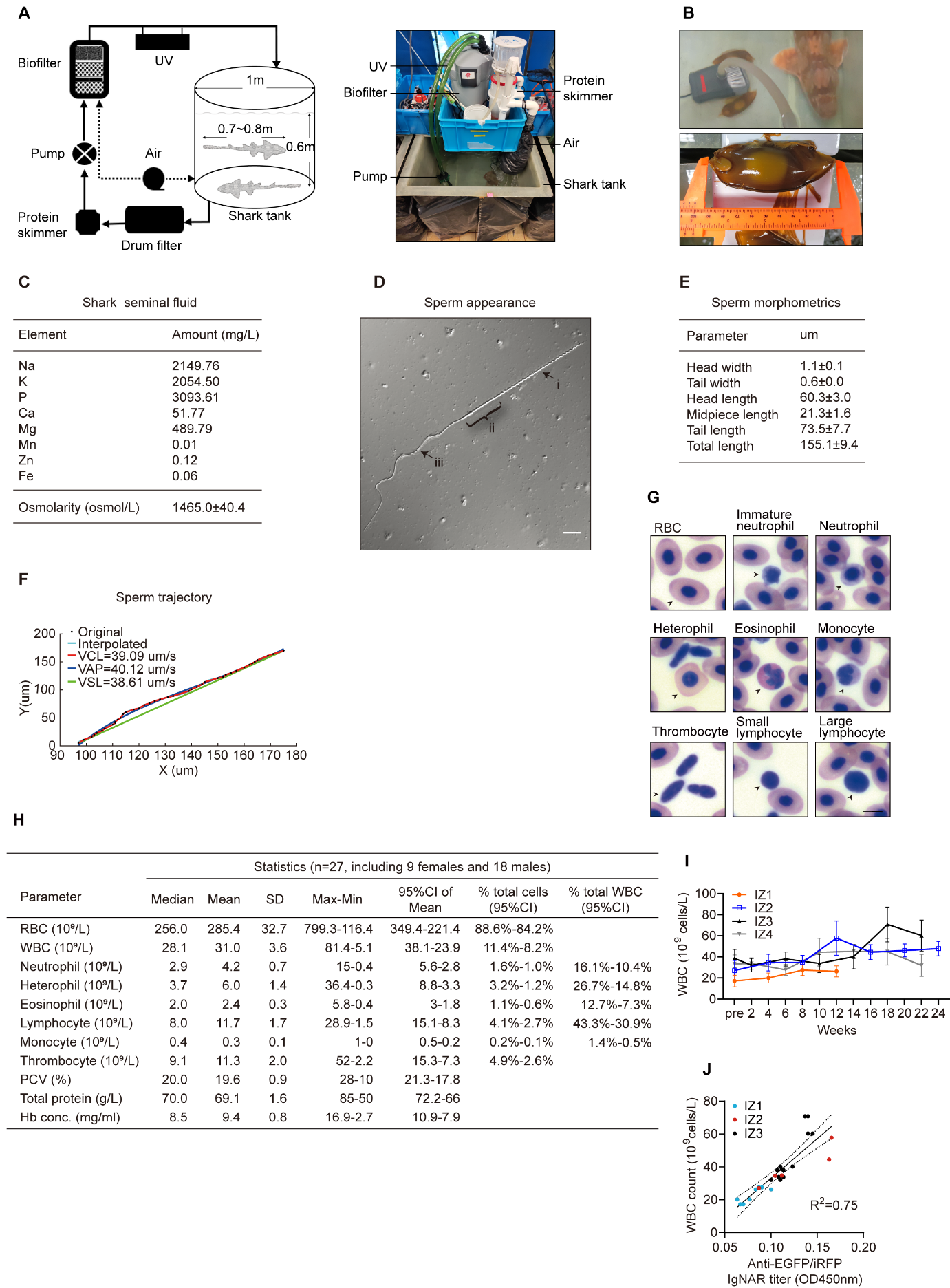


**Fig. S2. Aquarium-maintained bamboo sharks with reproduction potential and hematologic parameters and immune cell response of bamboo shark during immunization.** (**A**) Design of small aquarium for bamboo sharks*.* (**B**) Bamboo shark eggs laid in the system. (**C**) Element concentration in bamboo shark seminal fluid and its osmolarity. (**D**) Microscopic examination of bamboo shark sperms. i, head; ii, midpiece; iii, tail. Scale bar = 10 µm. (**E**) Sperm morphometrics. (**F**) Sperm trajectories. VCL, curvilinear velocity (i.e., actual velocity along the trajectory); VAP, average path velocity (i.e., velocity along a derived smoothed path); VSL, straight line velocity (i.e., straight-line distance between the starting and end points) of the track divided by the time of the track. The interpolated line is a line to connect the data values. (**G**) Wright–Giemsa-stained peripheral blood cells of bamboo sharks*.* Scale bar = 10 µm. Arrowheads indicate the cell type as described in the panel title. (**H**) Hematologic parameters of healthy adult bamboo sharks (n=27) prior to immunization. RBC, red blood cell; WBC, white blood cell; PCV, packed cell volume; Hb conc., hemoglobin concentration; SD, standard deviation; CI, confidential interval. (**I**) Changes in WBC count of bamboo sharks during immunization (n=3). White blood cells (WBC) consist of neutrophil, eosinophil, heterophil, monocyte, and lymphocyte. Blood samples were from three individuals for each immunization program. (**J**) Linear correlation analysis between anti-GFP/iRFP713 IgNAR level and WBC count. Data (n=29) were from three immunization programs (IZ1, IZ2, and IZ3).


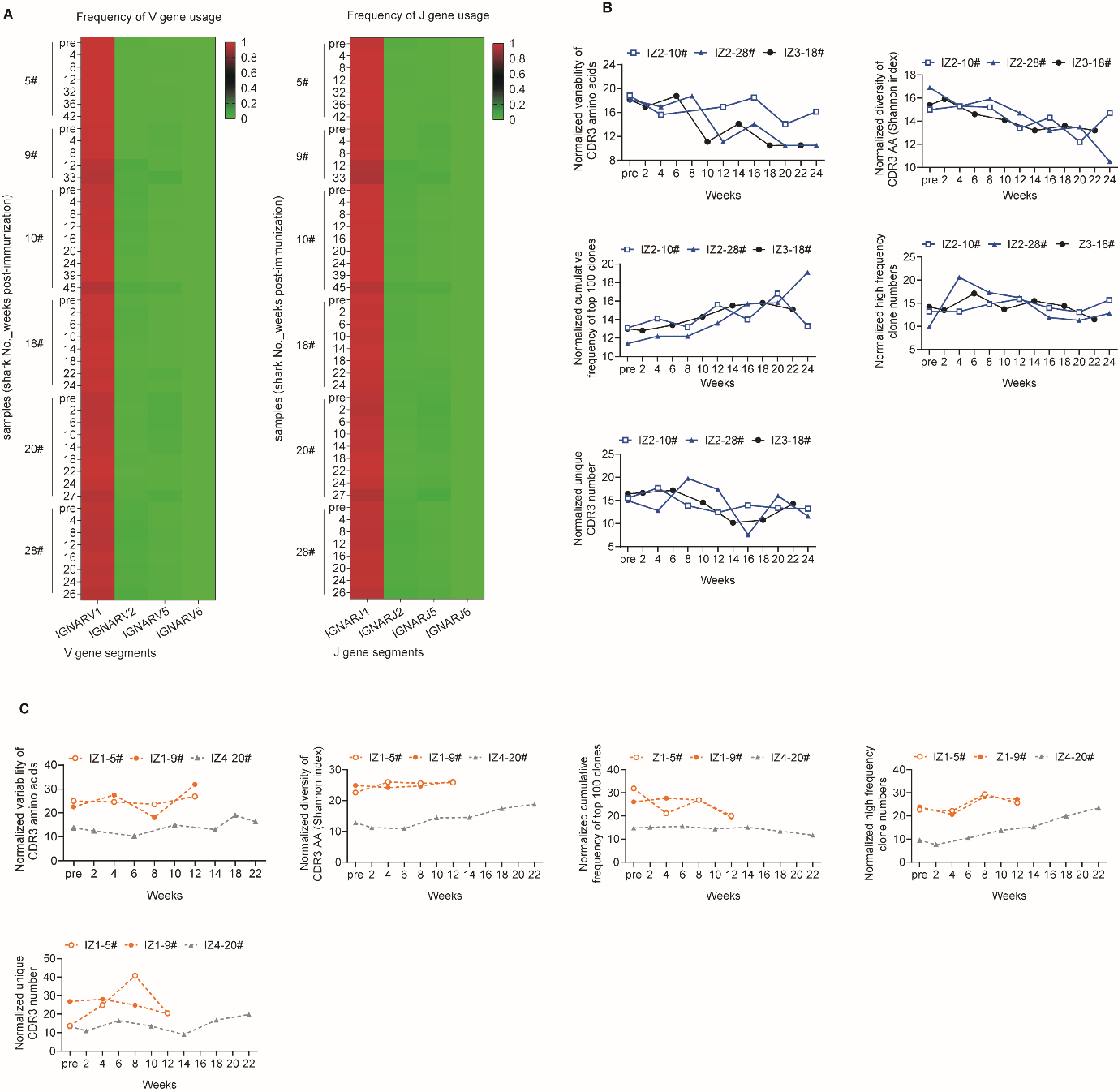


**Fig. S3. Profile of individual V gene and J gene usage and characteristics of vNAR immune repertoire during immunization progression.** (**A**) Usage development of individual V and J genes of four IgNAR clusters in the vNAR repertoire of six bamboo sharks. Heat map shows the V/J gene usage proportion. Each row corresponds to a sample; each column corresponds to a V/J germline gene; the color in each tile corresponds to the frequency of V/J gene in the repertoire. (**B**) Variability and diversity (Shannon index) of CDR3, cumulative frequency of top 100 clones, and high-frequency (>0.1%) clone numbers and unique CDR3 clone number in the vNAR repertoire of sharks in IZ2 and IZ3 during immunization. (**C**) Variability and diversity (Shannon index) of CDR3, cumulative frequency of top 100 clones, and high-frequency (>0.1%) clone numbers and the unique CDR3 clone number in the vNAR repertoire of sharks in IZ1 and IZ4 during immunization.


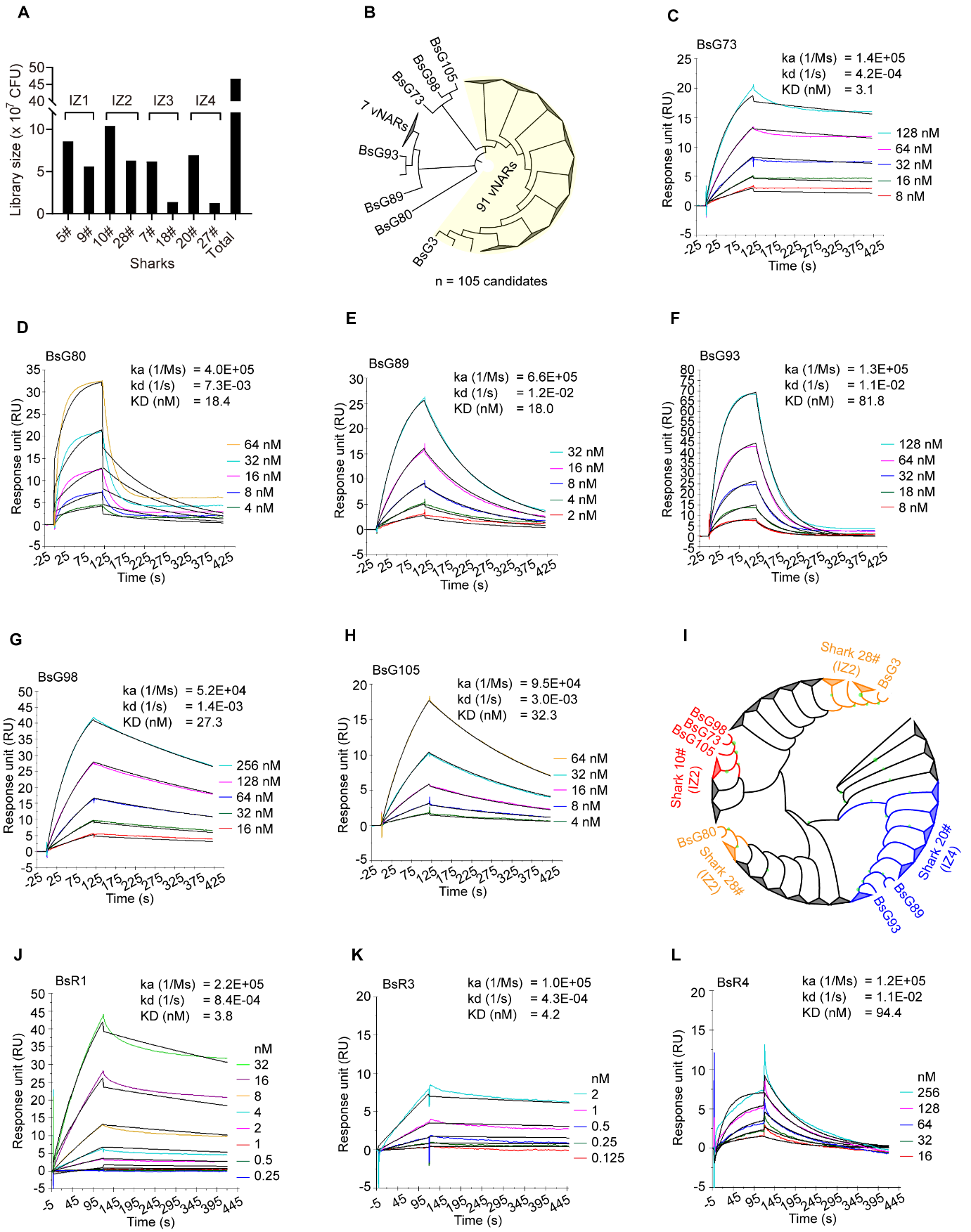


**Fig. S4. Phylogenetic analysis and binding affinity determination of GFP-specific and iRFP713-specific vNAR candidates.** (**A**) Composition of the phage library. (**B**) Phylogenetic analysis of 105 anti-GFP vNAR candidates from biopanning. The finally selected seven unique anti-GFP vNARs were indicated. (**C** to **H**) SPR sensorgrams of six vNARs binding with GFP. (**I**) Phylogenetic tree of bamboo shark vNARs (n=407 sequences) indicates the shark origin of seven unique anti-GFP vNARs. The 400 of 407 sequences were the pre-panning vNAR sequences from eight immunized sharks used for vNAR-phage library construction; the other sequences (n=7) were GFP-specific vNAR sequences. Except for the black clades, the clade with the same color denotes the vNARs from the same immunization program. (**J** to **L**) SPR sensorgrams of three vNARs binding with iRFP713.


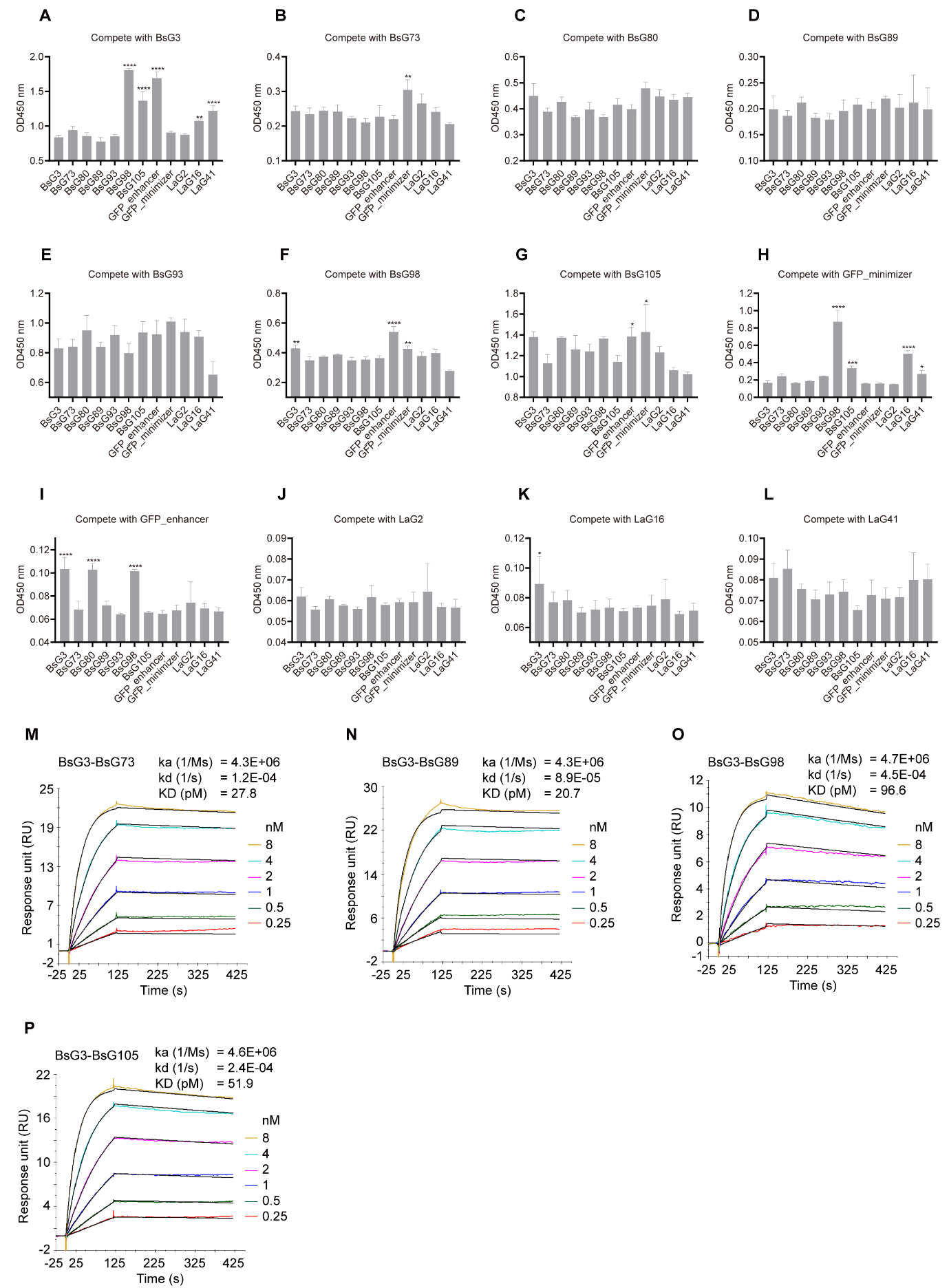


**Fig. S5. Overlapping/non-overlapping epitopes prediction of GFP-specific sdAbs and determination of binding affinities of bivalent vNARs to GFP.** (**A** to **L**) Overlapping/non-overlapping epitope prediction of 12 anti-GFP sdAbs by competitive epitope binding assay. Three repeats were performed for each value. Significant level (*P*<0.05) was marked as an asterisk. GFP_enhancer (64), GFP_minimizer (64), LaG2 (37), LaG16 (37), and LaG41 (37) were VHHs. (**M** to **P**) SPR sensorgrams of four bivalent vNARs binding with GFP.

**Table S1. NGS initial processing output of IgNAR immune repertoire.**


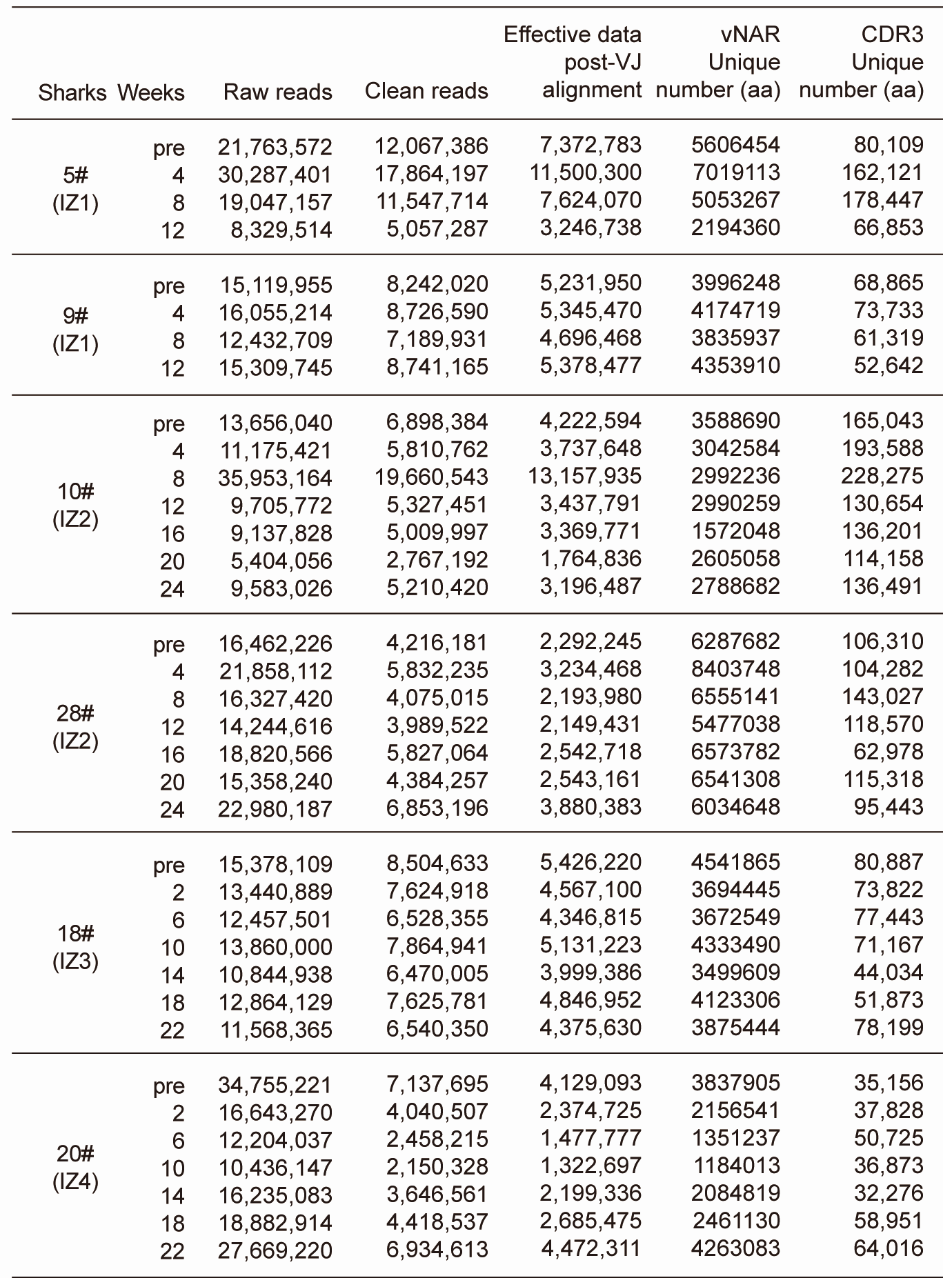


**Table S2. NGS initial processing output of phage-display vNAR library.**


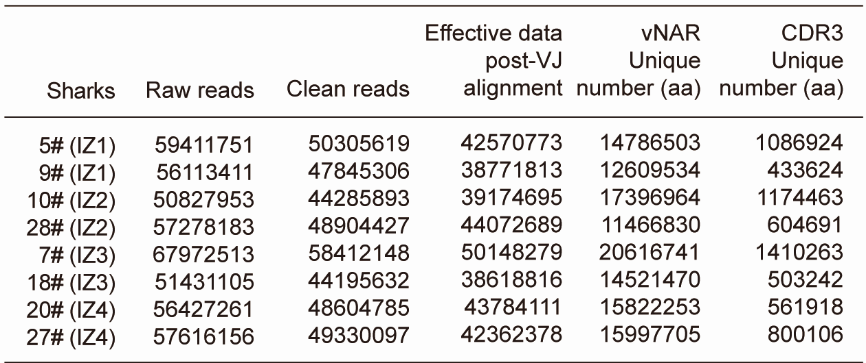

Supplement: Supplementary file 1 [file DataSheet1.DOCX]
